# Novel radiopaque ethanol injection: physicochemical properties, animal experiments, and clinical application in vascular malformations

**DOI:** 10.1186/s40779-024-00542-7

**Published:** 2024-06-20

**Authors:** Yu-Chen Shen, De-Ming Wang, Xi-Tao Yang, Zhen-Feng Wang, Ming-Zhe Wen, Yi-Feng Han, Lian-Zhou Zheng, Ruo-Yu Di, Chun-Yu Jiang, Jing-Bing Wang, Jian-Xiong You, Li-Ming Zhang, Li-Xin Su, Xin-Dong Fan

**Affiliations:** Vascular Anomaly Center, Department of Interventional Therapy, Shanghai Ninth People’s Hospital, Shanghai Jiao Tong University School of Medicine, Shanghai, 200011 China

**Keywords:** Vascular malformation, Ethanol, Sclerotherapy, Radiopacity, Iopromide, Radiopaque ethanol injection

## Abstract

**Background:**

Despite the efficacy of absolute ethanol (EtOH), its radiolucency introduces several risks in interventional therapy for treating vascular malformations. This study aims to develop a novel radiopaque ethanol injection (REI) to address this issue.

**Methods:**

Iopromide is mixed with ethanol to achieve radiopacity and improve the physicochemical properties of the solution. Overall, 82 male New Zealand white rabbits are selected for in vivo radiopacity testing, peripheral vein sclerosis [animals were divided into the following 5 groups (*n* = 6): negative control (NC, saline, 0.250 ml/kg), positive control (EtOH, 0.250 ml/kg), low-dose REI (L-D REI, 0.125 ml/kg), moderate-dose REI (M-D REI, 0.250 ml/kg), and high-dose REI (H-D REI 0.375 ml/kg)], pharmacokinetic analyses (the blood sample was harvested before injection, 5 min, 10 min, 20 min, 40 min, 1 h, 2 h, 4 h, and 8 h after injection in peripheral vein sclerosis experiment), peripheral artery embolization [animals were divided into the following 5 groups (*n* = 3): NC (saline, 0.250 ml/kg), positive control (EtOH, 0.250 ml/kg), L-D REI (0.125 ml/kg), M-D REI (0.250 ml/kg), and H-D REI (0.375 ml/kg)], kidney transcatheter arterial embolization [animals were divided into the following 4 groups (*n* = 3): positive control (EtOH, 0.250 ml/kg), L-D REI (0.125 ml/kg), M-D REI (0.250 ml/kg), and H-D REI (0.375 ml/kg); each healthy kidney was injected with saline as negative control], and biosafety evaluations [animals were divided into the following 5 groups (*n* = 3): NC (0.250 ml/kg), high-dose EtOH (0.375 ml/kg), L-D REI (0.125 ml/kg), M-D REI (0.250 ml/kg), and H-D REI (0.375 ml/kg)]. Then, a prospective cohort study involving 6 patients with peripheral venous malformations (VMs) is performed to explore the clinical safety and effectiveness of REI. From Jun 1, 2023 to August 31, 2023, 6 patients [age: (33.3 ± 17.2) years] with lingual VMs received sclerotherapy of REI and 2-month follow-up. Adverse events and serious adverse events were evaluated, whereas the efficacy of REI was determined by both the traceability of the REI under DSA throughout the entire injection and the therapeutic effect 2 months after a single injection.

**Results:**

The REI contains 81.4% ethanol (v/v) and 111.3 mg/ml iodine, which can be traced throughout the injection in the animals and patients. The REI also exerts a similar effect as EtOH on peripheral venous sclerosis, peripheral arterial embolization, and renal embolization. Furthermore, the REI can be metabolized at a similar rate compared to EtOH and Ultravist® and did not cause injury to the animals’ heart, liver, spleen, lungs, kidneys and brain. No REI-related adverse effects have occurred during sclerotherapy of VMs, and 4/6 patients (66.7%) have achieved complete response at follow-up.

**Conclusion:**

In conclusion, REI is safe, exerts therapeutic effects, and compensates for the radiolucency of EtOH in treating VMs.

**Trial registration:**

The clinical trial was registered as No. ChiCTR2300071751 on May 24 2023.

**Supplementary Information:**

The online version contains supplementary material available at 10.1186/s40779-024-00542-7.

## Background

Vascular malformations are congenital diseases caused by developmental defects in blood or lymphatic vessels [1]. Clinically, ethanol has been used in the endovascular therapy of vascular malformations for nearly four decades [2]. In our center, ethanol was first applied for managing peripheral arteriovenous malformations (AVMs) in 2009 [3, 4]. We have reported a series of studies on peripheral AVMs with promising outcomes of ethanol embolotherapy [5–8]. For venous malformations (VMs), the most widely used agents include ethanol, polidocanol, lauromacrogol, and bleomycin [9]. Among these, absolute ethanol (EtOH) is the most effective, with the lowest recurrence rate [10]. Our previous study also confirmed the efficacy of ethanol in treating complex head and neck VMs [11]. However, despite its satisfactory prognosis, ethanol is a double-edged sword. Given the radiolucency of ethanol, clinicians have to perform a blind injection under digital subtraction angiography (DSA) because of the danger of diffusion beyond the target [12]. The unawareness of injection speed and flow direction increases the risk of ethanol reflux into the normal vasculature. Improper injection of high-concentration ethanol can result in serious complications, including necrosis, nerve injury, cardiopulmonary accidents, and even death [13, 14].

Previous studies have attempted to mix iohexol or lipiodol with ethanol to render the solution radiopaque [15, 16]. However, despite the satisfactory results, follow-up reports and clinical trials are lacking, and no mature products have been launched. Ethanol injection at a concentration of > 80% (v/v) was reported to be effective in treating vascular malformations [17], but the above studies did not achieve sufficient ethanol concentrations (both < 75%). Theoretically, an ideal radiopaque ethanol injection (REI) should meet the following requirements: 1) be visible under DSA, 2) minimize the patient’s iodine intake, 3) maximize the effective ethanol content, and 4) have excellent stability. There is an urgent need to determine the optimal contrast media to balance the radiopacity at high concentrations.

The current work introduces a novel REI compensating the radiolucency of EtOH in the interventional treatment of vascular malformations. In the pre-exploration stage, the REI was prepared with a high concentration of ethanol and was stable. Based on its radiopacity, we controlled the iopromide concentration of the REI to reduce iodine intake in vivo. Pharmacodynamic and pharmacokinetic experiments were then systematically conducted in the animals. Finally, a cohort of patients with VMs was enrolled to receive sclerotherapy using the novel REI. This study aimed to evaluate REI’s efficacy and safety systematically.

## Materials and methods

### Preparation of REI

REI used for both animals and patients of the present work was prepared by mixing the clinically available iopromide and ethanol. To prepare an REI with a total volume of 100 L, 11.0 kg water for injection (WFI) was first heated to 90 ℃, and then 22.9 kg iopromide raw material powder (Brilliant Pharmaceutical Co., Ltd., Chengdu, China) was slowly added into the heated WFI, stirring until the powder was completely dissolved. The liquid was subsequently cooled to < 50 ℃, and 63.2 kg EtOH was added, stirring evenly. The whole solution was then filtrated using a 0.45-μm or 0.22-μm polytetrafluoroethylene filter. Finally, the REI was filled into each vial with 10 ml and sterilized at 121 ℃ for 15 min.

Viscosity measurement, purity testing, ethanol and iopromide content detection of REI are detailed in Additional file 1: Materials and methods.

### Animals

The animal study was approved by the Experimental Animal Management and Animal Ethics and Welfare Committee of Shanghai Mincal Medical Research Co., Ltd. [(2022) 07–25]. All animal experiments were designed and conducted according to the Arrive Guidelines 2.0 (https://arriveguidelines.org/arrive-guidelines).

Invasive procedures (blood collection, injection, and angiography) were performed under general anesthesia with endotracheal intubation. Ibuprofen was used for postoperative analgesia, and cefoxitin sodium was used for preventing postoperative infection.

A total of 82 male New Zealand white rabbits (approximately 3 months old) weighing 2.98–4.12 kg were included in the experiment (Hongfeng Rabbit Farm, Fuyang City, Zhejiang Province, China). Anesthesia was induced by intramuscular injection of Zoletil®50 (Virbac, Carros, France) and xylazine, followed by endotracheal intubation (2.5 caliber). Continuous inhalation anesthesia with isoflurane or sevoflurane was administered during surgery. Ultravist® 300 (Bayer, Leverkusen, Germany) was used in all angiographies and contrast-enhanced computed tomographies (CTs).

### Detection of in vitro and in vivo radiopacity

For in vitro radiopacity measurements, the samples were analyzed by X-ray transmission (INFX-9000C, Canon, Japan). One rabbit was selected for in vivo radiopacity testing. A 24-G indwelling needle was placed in the bilateral auricular veins for baseline angiography and sample injection under DSA. The right groin skin and subcutaneous tissue were cut, the right femoral artery was exposed, and a 4F vascular sheath (Terumo Corporation, Tokyo, Japan) was placed. A 4F guiding catheter (Cook Medical, Bloomington, IL, USA) was used to perform angiography of the abdominal aorta, and a schematic map was generated to indicate the route to the renal artery. Next, a 2.5F microcatheter (Cook Medical, Bloomington, IL, USA) was inserted into the renal artery for baseline angiography and subsequent sample injections using DSA. Finally, all images were recorded.

### Endovascular sclerosis of peripheral vein

Peripheral vein sclerosis experiments were performed in 31 rabbits, and 1 (3.2%) died due to anesthetic overdose. The animals were divided into the following 5 groups (*n* = 6): negative control (NC, 0.250 ml/kg), positive control (EtOH, 0.250 ml/kg), low-dose (L-D) REI (0.125 ml/kg), moderate-dose (M-D) REI (0.250 ml/kg), and high-dose (H-D) REI (0.375 ml/kg). Saline was used as an NC, and EtOH (Sinopharm Guorui Pharmaceutical Co., Ltd., Anhui, China) was used as a positive control. Baseline DSA was performed using a 22-G butterfly needle inserted through the auricular central artery. A 24-G indwelling needle was placed in the auricular veins (Additional file 1: Fig. S1a) for sample injection (20 ml/h through a micropump; Additional file 1: Fig. S1b). DSA was re-examined 14 d postoperatively. The devascularization rate was calculated based on the area of the blood vessels. The appearance of rabbit ears was recorded before embolization and at 3, 7, and 14 d postoperatively. All rabbits were sacrificed at 14 d postoperatively. The auricular vein and perivenous tissue of both the normal and embolized ears were harvested for histopathological analyses. The standards for sclerotic grading are presented in Additional file 1: Table S1.

For the subsequent pharmacokinetic analyses, 20 ml of blank blood was harvested preoperatively. The blood samples were simultaneously collected through the auricular vein of the normal side at the following time points: 5 min, 10 min, 20 min, 40 min, 1 h, 2 h, 4 h, and 8 h after injection. All blood samples for pharmacokinetics were immediately stored in EDTA-containing vacuum collection vessels and centrifuged at 1800 × *g* for 10 min at 4 °C to obtain plasma. The plasma was equally divided into two aliquots, placed into cryotubes, and immediately stored at –80 ℃ until analyses. Quantification of plasmatic ethanol and iopromide was detailed in Additional file 1: Materials and methods.

### Pharmacokinetic analyses

Phoenix WinNonlin Version 6.4 (Certara, NJ, USA) was used to calculate the primary pharmacokinetic parameters, including maximum concentration (C_max_), elimination half-life (T_1/2_), volume of distribution at steady state (Vdss), clearance, time of last observed concentration (T_last_), area under the curve (AUC; AUC_0-last_ and AUC_0-∞_), and mean residence time (MRT; MRT_0-last_ and MRT_0-∞_) of iopromide and ethanol, all of which were determined from plasmatic concentration–time profiles by non-compartmental analysis.

### Endovascular embolization of peripheral artery

Embolization of the peripheral artery was performed in 18 rabbits, 3 of which died due to anesthetic overdose (16.7%). The animals were divided into the following 5 groups (*n* = 3): NC (0.250 ml/kg), positive control (EtOH, 0.250 ml/kg), L-D REI (0.125 ml/kg), M-D REI (0.250 ml/kg), and H-D REI (0.375 ml/kg). Saline was used as an NC, and EtOH (Sinopharm Guorui Pharmaceutical Co., Ltd., Anhui, China) was used as a positive control. A 22-G butterfly needle was placed in the auricular central artery (Additional file 1: Fig. S1c) for angiography or sample injection (20 ml/h through a micropump, Additional file 1: Fig. S1b). Baseline angiography was performed before the injection, and DSA was re-examined 14 d postoperatively. The devascularization rate was calculated based on the area of the blood vessels. The appearance of rabbit ears was recorded before embolization and at 3, 7, and 14 d postoperatively. All the rabbits were sacrificed at 14 d postoperatively. The color of the embolized ear tissue was recorded, and the discolored area was measured. The auricular central artery, periarterial tissue, and distal tissue of both normal and embolized ears were harvested for histopathological analyses. The embolic grading standards are detailed in Additional file 1: Tables S2-S4.

### Transcatheter arterial embolization (TAE) of the kidneys

TAE experiments on the kidneys were performed in 16 rabbits, 4 of which died after injection (25.0%). The animals were divided into the following 4 groups (*n* = 3): positive control (EtOH, 0.250 ml/kg), L-D REI (0.125 ml/kg), M-D REI (0.250 ml/kg), and H-D REI (0.375 ml/kg). Each healthy kidney was injected with saline. Ethanol (Sinopharm Guorui Pharmaceutical Co., Ltd., Anhui, China) was used as the positive control. First, the skin and subcutaneous tissues of the right groin were incised. Then, the right femoral artery was exposed, and a 4F vascular sheath (Terumo Corporation, Tokyo, Japan) was placed (Additional file 1: Fig. S1d). A 4F guiding catheter (Cook Medical, Bloomington, IL, USA) was used to perform angiography of the abdominal aorta, and a schematic map was generated to indicate the route to the renal artery.

Next, we inserted a 2.5F microcatheter (Cook Medical, Bloomington, IL, USA) into the renal artery for baseline angiography and subsequent sample injection (20 ml/h through a micropump, Additional file 1: Fig. S1b). Renal angiography was performed before and 2 h after injection. The right femoral artery was ligated postoperatively. The abdominal aortography was performed through the left femoral artery under DSA on the 21st day postoperatively. The devascularization rate was calculated based on the renal angiogram area. Subsequently, contrast-enhanced CT (TSX-303A; Canon, Japan) of the abdominal aorta was performed. A three-dimensional reconstruction of the kidney was performed using the Vitrea Advanced Visualization Platform (Canon, Tokyo, Japan) to determine the effective volume of the kidney. All rabbits were sacrificed after the CT examinations. The renal arteries and tissues of the normal and embolized sides were harvested for histopathological analysis.

### Safety evaluation of REI

Overall, 16 rabbits were selected for the safety evaluation of the REI, and 1 rabbit (6.3%) died due to anesthetic overdose. The animals were divided into the following 5 groups (*n* = 3): NC (0.250 ml/kg), H-D EtOH (0.375 ml/kg), L-D REI (0.125 ml/kg), M-D REI (0.250 ml/kg), and H-D REI (0.375 ml/kg). Saline was used as an NC. Venous and arterial blood were collected from the normal ear at the following points of time: pre-injection, 12 h, 24 h, 3 d, 7 d, 14 d, 21 d, and 28 d after injection.

Venous blood was examined for routine blood tests (BC-5000VET, Mindray, Shenzhen, China), biochemical assays (BS-240VET, Mindray, Shenzhen, China) and myocardial zymogram analyses (Synergy-2, BioTek, USA), and arterial blood was examined for blood gas analysis (GEM4000, Werfen, Barcelona, Spain). Animals’ alanine aminotransferase (ALT), aspartate transaminase (AST), alkaline phosphatase (ALP), serum creatinine, blood urea, creatine kinase-MB (CKMB), α-hydroxybutyrate dehydrogenase (HBDH), partial pressure of oxygen (pO_2_), white blood cell (WBC), and hemoglobin (HGB) levels were all focused. All animals were sacrificed after 28 d, and the heart, liver, spleen, lungs, kidneys, and brain were harvested for histopathological analyses.

### Histopathological analyses

The tissues used for paraffin sectioning were immersed in 4% paraformaldehyde (Biosharp, Anhui, China) for 48 h and then transferred to 70% ethanol. Individual lobes of tissue biopsy material were placed in processing cassettes, dehydrated using a serial alcohol gradient, and embedded in paraffin wax blocks. Before staining, 3-μm-thick sections were dewaxed in xylene, deparaffinized, and rehydrated in distilled water.

#### HE staining

The slides were stained with hematoxylin (RecordBio, Shanghai, China) for 5–10 min, washed with tap water, differentiated with 1% hydrochloric acid alcohol for several seconds, and rinsed with tap water. The slides were then turned blue using 1% ammonia water and rinsed with running water. Finally, the slides were placed in an eosin dye solution (RecordBio, Shanghai, China) and stained for 5–10 min.

#### Masson’s trichrome staining

The slices were immersed overnight in potassium dichromate and washed with running water. Thereafter, the slices were immersed in an iron hematoxylin dye solution, soaked for 1 min, washed with tap water, differentiated with 1% hydrochloric acid alcohol, and washed with tap water. Then, they were soaked in Ponceau red acid fuchsin solution for 6 min and rinsed with tap water. Finally, the slices were stained with 1% phosphomolybdate solution for 1 min. Without washing, the slides were slightly drained and dyed directly with a 2.5% aniline blue solution for 30 s. The slices were then rinsed, differentiated with 1% glacial acetic acid, and dehydrated in two tanks containing anhydrous ethanol.

#### Periodic acid-Schiff staining

The slides were oxidized in a 0.5% periodic acid solution for 5 min and then rinsed with distilled water. Subsequently, the slides were placed in Schiff’s reagent (RecordBio, Shanghai, China) for 15 min and then washed with lukewarm tap water for 5 min. The slides were counterstained with Mayer’s hematoxylin for 1 min and washed in tap water for 5 min.

#### Sirius red staining

The tissue sections were completely covered with picro Sirius red solution (RecordBio, Shanghai, China) and incubated for 60 min. The slides were then rinsed quickly with two changes of acetic acid solution. Next, the slides were rinsed in absolute alcohol and dehydrated with two changes of absolute alcohol.

#### Toluidine blue staining

The sections were stained with a toluidine blue working solution (RecordBio, Shanghai, China) for 2–3 min and washed thrice in distilled water with 3 changes. The slides were then dehydrated quickly using 95% ethanol and two changes of 100% ethanol. Finally, all the slides were cleared in xylene and mounted with neutral balsam.

### Immunofluorescence

Terminal deoxynucleotidyl transferase-mediated dUTP nick-end labeling (TUNEL) immunofluorescence was used to detect apoptosis. In brief, the tissue was covered with protease K (RecordBio, Shanghai, China) working solution (protease K storage solution diluted with PBS in a 1:9 ratio) and incubated at 37 °C for 30 min. The slides were then washed thrice with PBS for 5 min each time, after which the slides were incubated with Triton X-100 (RecordBio, Shanghai, China) for 20 min and washed three times with PBS for 5 min each time. The slides were covered with TDT and dUTP and incubated at 37 °C for 2 h, following the in situ cell death detection kit POD (11684817910, Roche, Switzerland) instructions. DAPI was applied to dye the nucleus. Finally, the slides were mounted with an anti-fade mounting medium.

### Transmission electron microscopy (TEM) examination

The tissue sample used for TEM was immediately immersed in 2% tissue fixative, which was made by mixing 4% polyformaldehyde (Shuzbio, Shanghai, China) with the same volume of 4% glutaraldehyde (Titan Scientific, Shanghai, China), at 4 ℃ for 12 h. The sample was then rinsed with 0.1 mol/L phosphoric acid buffer (15 min × 3 times) and fixed with 1% osmic acid solution for 1–2 h. The samples were rinsed again with a phosphoric acid buffer solution. Next, dehydration was performed using an ethanol solution of gradient concentrations (30%, 50%, 70%, 80%, 90%, 95%, and 100%). Finally, the sample was treated twice with pure acetone for 10 min each time, a mixture of embedding agent and acetone (v/v = 1/3) for 1 h; a mixture of embedding agent and acetone (v/v = 1/1) for 3 h; a mixture of embedding agent and acetone (v/v = 3/1) for 10 h; and a pure embedding agent for 24 h. The permeated samples were embedded and polymerized at 37 ℃, 45 ℃, and 60 ℃ for 72 h. The samples were then cut into 70–90 nm sections (EM UC7, Leica, Germany), and the slices were double-stained with a lead citrate solution and uranium acetate 50% ethanol saturated solution for 5–10 min. Finally, the slices were observed by TEM (HT7700, Hitachi, Japan).

### Clinical application of REI

#### Ethics approval and registration of clinical trial

The clinical trial was designed and conducted according to the STROBE guideline (https://www.strobe-statement.org/). A cohort of patients with lingual VMs was enrolled to investigate the safety and therapeutic efficacy of REIs. This prospective clinical trial was approved by the Institutional Review Board of Shanghai Ninth People’s Hospital (SH9H-2023-T132-2) and was conducted according to the tenets of the Declaration of Helsinki. The study was registered in the Chinese Clinical Trial Registry (www.chictr.org.cn; No. ChiCTR2300071751). All patients provided written informed consent prior to study participation and treatment.

#### Patients and therapeutic procedures

From Jun 1, 2023 to August 31, 2023, 6 patients with lingual VMs [3 females and 3 males, average (range) age: 33.3 ± 17.2 (19–58) years] received sclerotherapy of REI. The first patient was selected as a sentinel subject. The inclusion and exclusion criteria are described in detail in Additional file 1: Materials and methods. All patients complained of VM lesions affecting their normal oral function. They were informed of the risks in detail. The protocol for REI sclerotherapy and evaluation of its safety and effectiveness are detailed in Additional file 1: Materials and methods. All patients were followed up for 2 months postoperatively.

### Statistical analyzes

Data are presented as mean ± standard deviation (SD). The devascularization rate and histopathological staining were quantified using ImageJ 1.53 k software (National Institutes of Health, USA). Three-dimensional reconstruction and volume estimation of VM lesions were completed using Mimics Medical 21.0 (Materialise, Leuven, Belgium). Comparisons among three or more groups were performed using one-way analysis of variance (one-way ANOVA). The Tukey test was used to conduct post-hoc analysis between every two groups. All statistical analyses were performed using SPSS (version 24.0; IBM Corp., Armonk, NY, USA) and Prism GraphPad 8.3 (GraphPad Software, San Diego, CA, USA). Statistical significance was set at *P* < 0.05.

## Results

### Physicochemical properties and radiopacity of REI

**Fig. 1:**
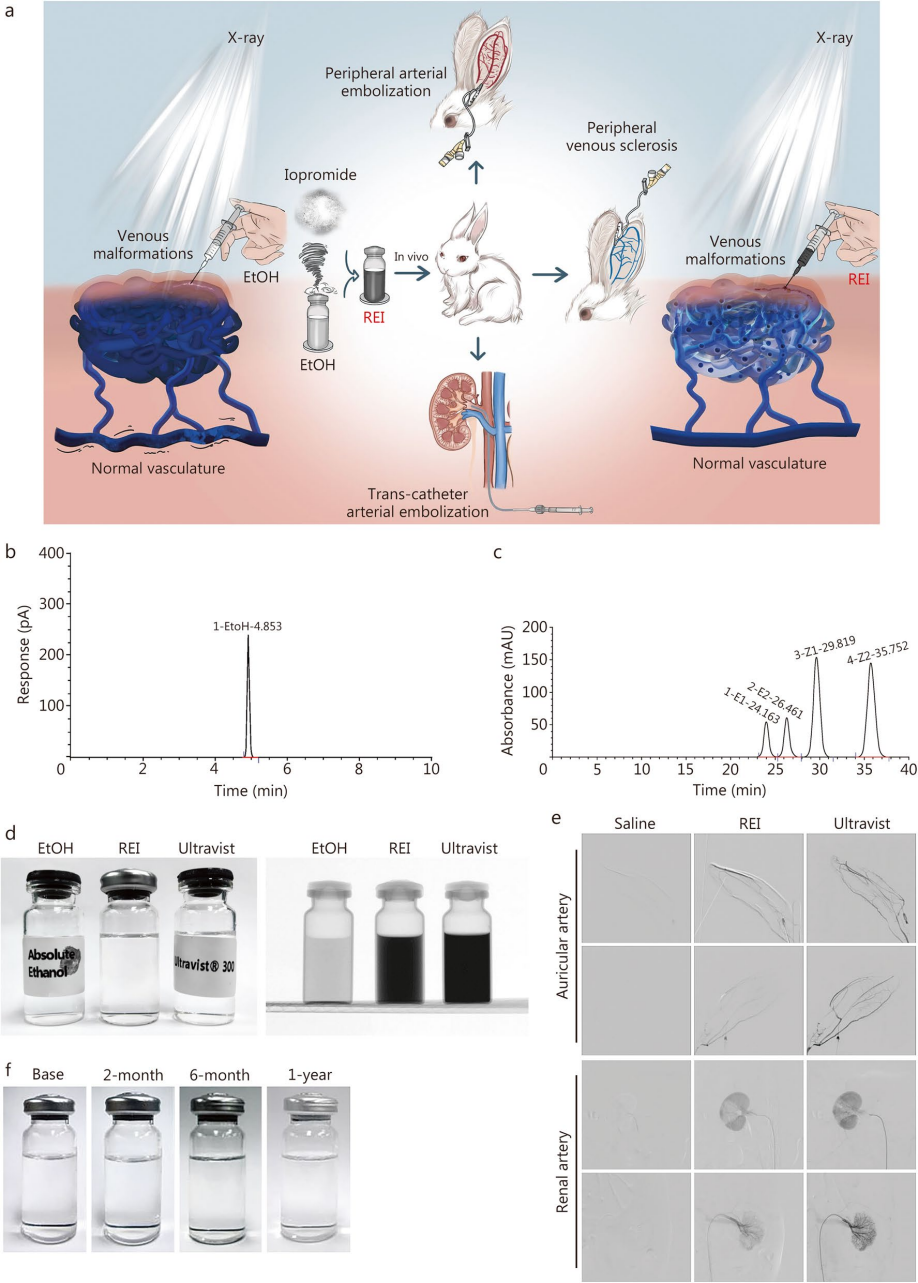
Physicochemical properties, radiopacity, and stability of radiopaque ethanol injection (REI). **a** Flow diagram of the present study. **b** High-performance gas chromatography (HPGC) examination of REI. **c** High-performance liquid chromatography (HPLC) examination of REI. **d** In vitro radiopacity of REI. **e** In vivo radiopacity of REI. **f** Long-term stability of REI in 25 ℃. Ultravist: Ultravist® 300 (Bayer, Leverkusen, Germany). EtOH absolute ethanol

The flow diagram of the present work is shown in Fig. 1a. The REI (total 10 ml per vial) contained 642.3 mg/ml ethanol [81.4% (v/v)] and 231.2 mg/ml iopromide, at an iodine concentration of 111.3 mg/ml. Ethanol existed with one configuration, whereas 4 configurations of iopromide were found in REI (Fig. 1b, c). The viscosity of REI was 3.6 mPa.s in 25 ℃ and 2.6 mPa.s in 37 ℃, whereas that of EtOH was 1.1 mPa.s in 25 ℃ and 0.8 mPa.s in 37 ℃ [18]. On high-performance liquid chromatography (HPLC), the purity of REI was 99.93%. The REI appeared as a dark image on DSA images in vitro (Fig. 1d). The REI solution can be traced in vivo when injected into the auricular and renal arteries (Fig. 1e). REI reflux was detected under DSA, suggesting excessive injection speed and pressure. A 1-year observation period demonstrated that the REI possessed reliable stability at room temperature (Fig. 1f).

### Efficacy of REI in peripheral venous sclerosis

**Fig. 2:**
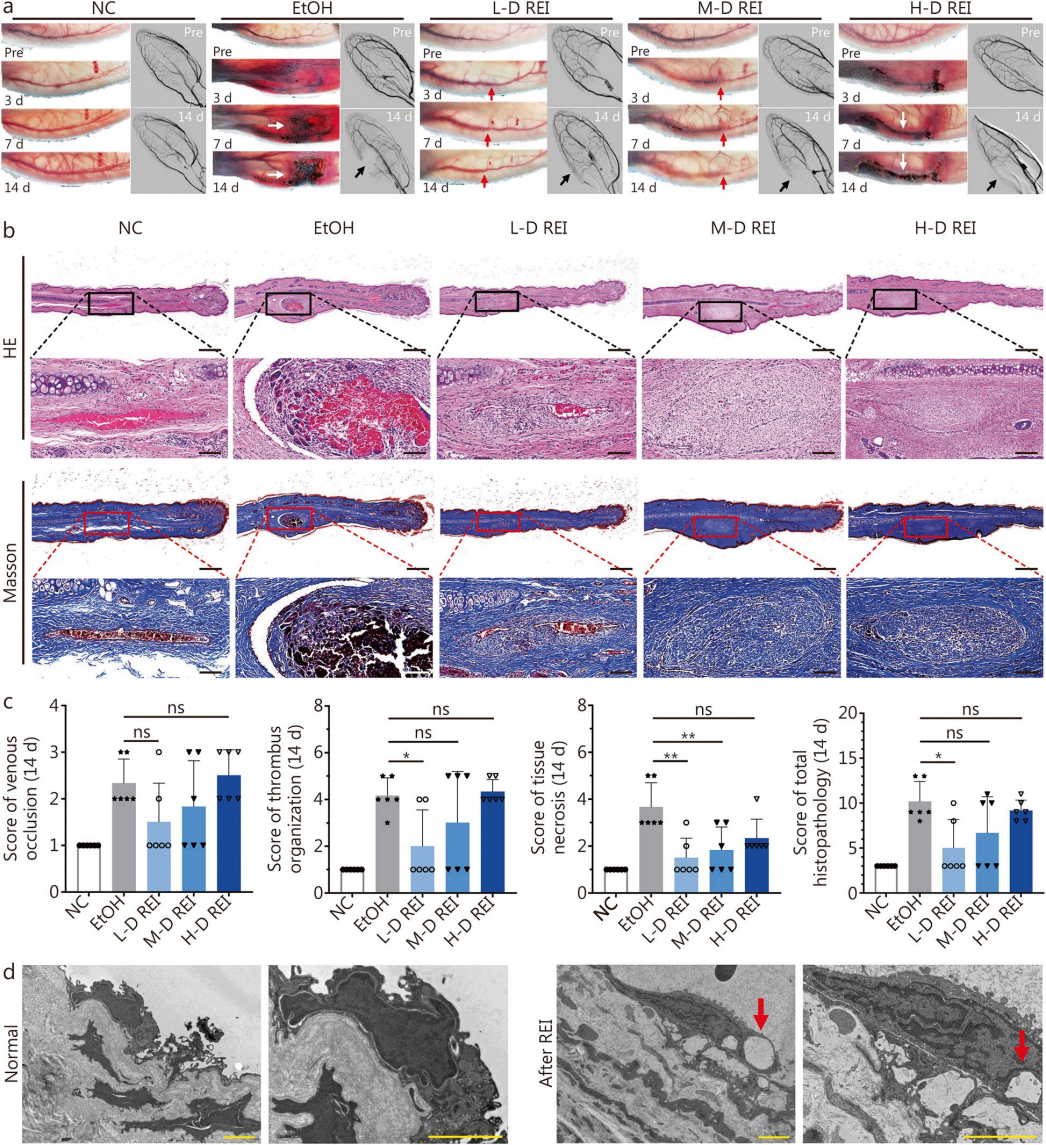
Sclerosis of auricular vein by radiopaque ethanol injection (REI). **a** General appearance and angiography of the ear after venous sclerosis. Red arrows indicate the occlusion of auricular vein; white arrows indicate the necrosis of perivascular tissue; black arrows indicate the devascularization on angiography. **b** Cross-sectional HE and Masson staining of sclerosis site of the ear. Scale bar = 1 mm (upper) or 100 μm (bottom). **c** Scores of venous occlusion (*P* = 0.0041), thrombus organization (*P* = 0.0004), tissue necrosis (*P* = 0.0001), and total histopathology (*P* = 0.0003) at the 14th day postoperatively (tested by One-Way ANOVA). **d** Transmission electron microscopy (TEM) examination of normal vein and vein after REI sclerosis. Red arrows indicate the vacuolar degeneration of venous endothelial cell. Scale bar = 2 μm. NC: 0.25 ml/kg saline; EtOH: 0.25 ml/kg EtOH; L-D REI: 0.125 ml/kg REI; M-D REI: 0.25 ml/kg REI; H-D REI: 0.375 ml/kg REI (*n* = 6). ^*^P < 0.05, ^**^P < 0.01 (Tukey test of post-hoc analysis). ns not significant, EtOH absolute ethanol, NC negative control, L-D low-dose, M-D moderate-dose, H-D high-dose, Pre preoperative

The auricular vein of the rabbit was selected to study peripheral vein sclerosis. During the 14-d observation period, REI caused dilation and tortuosity of the auricular vein (Fig. 2a, red arrows). Auricular angiography showed that the degree of vascular occlusion increased in an REI dose-dependent manner. Moreover, compared with EtOH (2/6, 33.3%), REI was associated with a lower rate of tissue necrosis, even with a high-dose injection (1/6, 16.7%; Fig. 2a; Additional file 1: Table S5).

Representative images of HE and Masson staining are shown in Fig. 2b. The score of venous occlusion, thrombus organization, and perivascular tissue necrosis increase along with the incremental dose of REI (Fig. 2c). No statistical differences are presented between each dose of REI and EtOH on the venous occlusion (*P* > 0.05, Fig. 2c). Although significant difference exists between the L-D REI group and the EtOH group on the thrombosis of lumen (*P* = 0.048), no significance is showed between the M-D/H-D REI group and the EtOH group (*P* > 0.05, Fig. 2c). Quantitative pathological analyses revealed discrepancy between the L-D REI group and the EtOH group (*P* = 0.014) but no significant difference between the M-D/H-D REI group and the EtOH group in the total score (*P* > 0.05); however, REI induced less tissue necrosis than EtOH at the same dose (*P* = 0.006, Fig. 2c). TEM revealed ultrastructural changes in the veins, including vacuolar degeneration of endothelial cells (red arrows) and the presence of venous intima (Fig. 2d), after sclerotherapy with REI.

### Efficacy of REI in peripheral arterial embolization

Given the difference in outcomes between venous and arterial occlusions, we used a different assessment system for embolization of the central auricular artery. The animals were divided into groups similar to those in the venous sclerosis experiment (*n* = 3). The experimental ears presented dynamic changes in tissue ischemia, further indicating the efficacy of the embolic agent. A significant difference in the score of auricular appearance is only found between the L-D REI group and the EtOH group 14 d after injection (*P* = 0.006; Additional file 1: Fig. S2a). Angiographic images showing the degree of devascularization at the 14th day endpoint are shown in Additional file 1: Fig. S2b, whereas quantitative analyses indicated no differences between EtOH and REI at equivalent doses (*P* > 0.05). Histopathological examination of the central auricular artery revealed a thrombus formation in the lumen. Furthermore, the distal ear tissue was prone to necrosis after arterial embolization, which indirectly reflected the efficacy of the embolic agent (L-D REI vs. EtOH: *P* = 0.039; M-D and H-D REI vs. EtOH: both *P* > 0.05; Additional file 1: Fig. S2c). Finally, the total score indicated that REI had a dose-dependent effect on arterial embolization and led to adequate vascular occlusion (L-D REI vs. EtOH: *P* = 0.015; M-D and H-D REI vs. EtOH: both *P* > 0.05; Additional file 1: Fig. S2c). TEM revealed that the ultrastructure of the arterial wall completely lost its normal morphology after REI embolization (Additional file 1: Fig. S2d).

### Efficacy of REI in TAE of the kidneys

Clinically, the main indications for renal artery embolization include pre-nephrectomy and pre-radiofrequency ablation infarction of renal tumors; palliations of unresectable renal malignancy; renal hemorrhage; and vascular malformations [19]. Experimentally, the kidney is the ideal organ for evaluating the efficacy of an embolic agent [20]. We performed TAE on rabbit kidneys to test the embolic potency of REI on organ. Renal angiography showed that the degree of renal vascular occlusion increased in an REI dose-dependent manner, and the embolic potency of REI can be compensated through dose enhancement (M-D REI vs. EtOH: *P* = 0.026; H-D REI vs. EtOH: *P* > 0.05; Additional file 1: Fig. S3a). Three-dimensional reconstruction of the kidney to assess the renal volume confirmed the angiographical results (M-D REI vs. EtOH: *P* < 0.0001; H-D REI vs. EtOH: *P* > 0.05; Additional file 1: Fig. S3b). Next, we carried out histopathological staining and TUNEL immunofluorescence of renal cross section. As shown in Additional file 1: Fig. S3c, despite the significant discrepancy between M-D REI group and EtOH group (*P* < 0.0001), H-D REI resulted in a similar degree of fibrosis as that of EtOH (*P* > 0.05). In addition, renal cells underwent an equivalent extent of apoptosis in the H-D REI and EtOH groups (*P* > 0.05; Additional file 1: Fig. S3d). Finally, ultrastructural analysis of the renal tissue revealed destruction of the nephrons after REI embolization (Additional file 1: Fig. S3e).

We also performed histological analysis of the renal artery at the injection site. As shown in Additional file 1: Fig. S4a, EtOH and REI effectively caused morphological abnormalities in the arterial lumen and led to the same extent of thrombosis (*P* > 0.05). However, the TUNEL immunofluorescence assay showed that REI had a better capacity to induce apoptosis than EtOH (M-D and H-D REI vs. EtOH: both *P* < 0.0001; Additional file 1: Fig. S4b). TEM examination indicated REI-induced damage to the endothelium and a disordered arrangement of smooth muscle cells (Additional file 1: Fig. S4c). These results added the reliability of REI’s embolic effectiveness.

### Pharmacokinetic parameters of REI

**Fig. 3:**
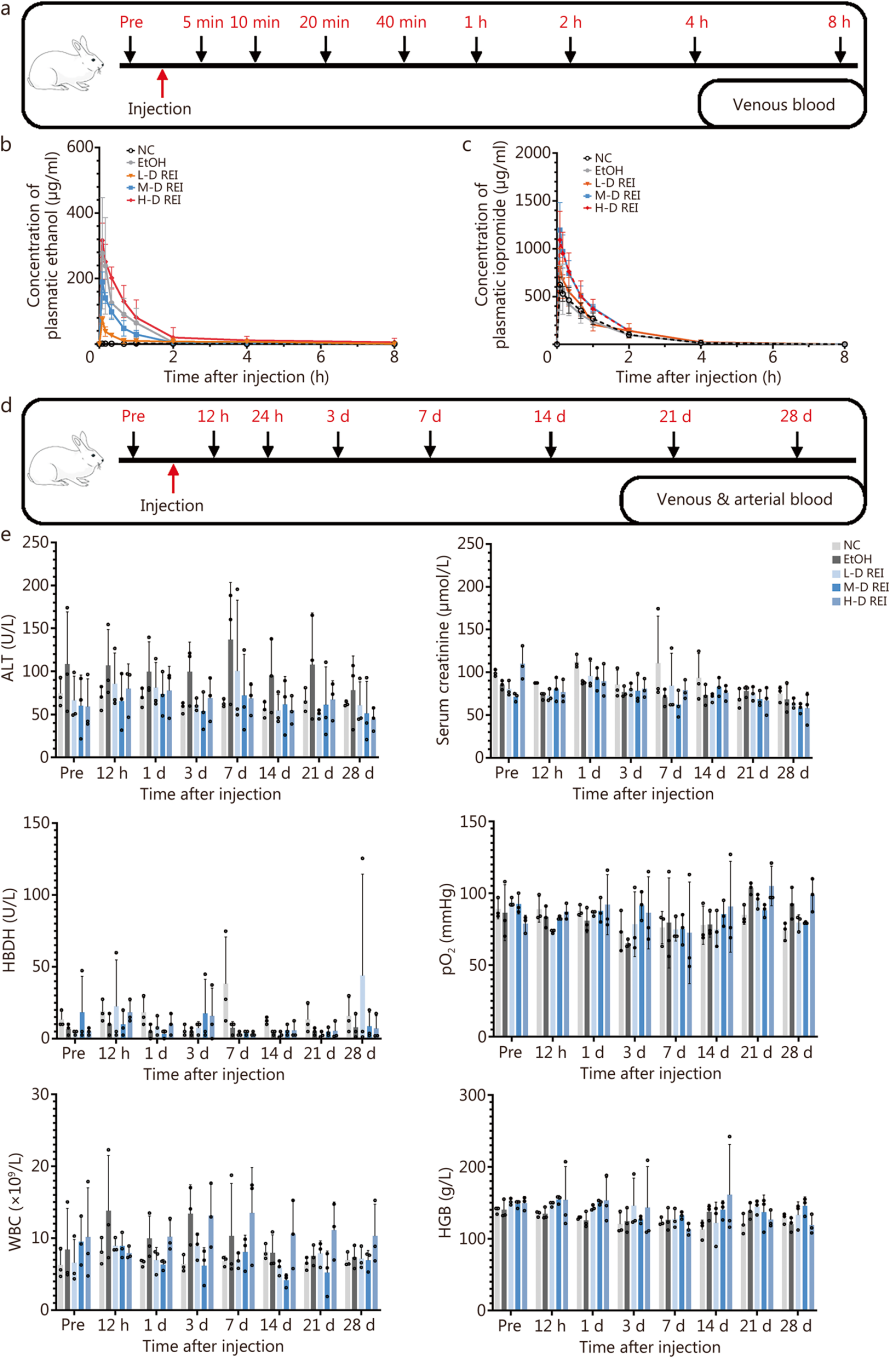
Pharmacokinetics and biosafety evaluation of radiopaque ethanol injection (REI) in rabbits. **a** Time points of blood collection for pharmacokinetic analysis (*n* = 6). **b** Plasmatic ethanol concentration–time curve. **c** Plasmatic iopromide concentration–time curve. **d** Time points of blood collection for safety evaluation (*n* = 3). **e** Dynamic changes in alanine aminotransferase (ALT), serum creatinine, α-hydroxybutyrate dehydrogenase (HBDH), partial pressure of oxygen (pO_2_), white blood cell (WBC), and hemoglobin (HGB) levels (*P* > 0.05; tested by one-way ANOVA at each time point). NC: 0.25 ml/kg saline; EtOH: 0.25 ml/kg EtOH; H-D EtOH: 0.375 ml/kg EtOH; L-D REI: 0.125 ml/kg REI; M-D REI: 0.25 ml/kg REI; H-D REI: 0.375 ml/kg REI. EtOH absolute ethanol, NC negative control, L-D low-dose, M-D moderate-dose, H-D high-dose, Pre preoperative

For pharmacokinetic analyses, venous blood was collected from the rabbits according to the time scale shown in Fig. 3a. Gas chromatography-triple quadrupole tandem mass spectrometry (GC–MS/MS) was used to quantify the plasma ethanol concentration. The plasma concentration–time profiles of ethanol were largely similar between the EtOH and H-D REI groups (Fig. 3b). The pharmacokinetic parameters of ethanol are detailed in Additional file 1: Table S6. The C_max_ gradually increased as the dose increased [(128.8 ± 23.1) μg/ml, (257.6 ± 66.2) μg/ml, and (399.4 ± 57.4) μg/ml for L-D, M-D, H-D REI groups, respectively]. However, clearance fluctuated in the groups; the T_1/2_ was similar between the EtOH group [(1.4 ± 0.6) h] and the H-D REI group [(1.2 ± 0.8) h]. Consistent with that of EtOH, the ethanol component of REI was completely metabolized within 8 h.

On LC–MS/MS, the plasma concentration–time profiles of iopromide were largely similar across groups (Fig. 3c). As shown in Additional file 1: Table S7, the C_max_ gradually increased as the dose increased [(1307.5 ± 747.8) μg/ml, (2527.0 ± 1275.0) μg/ml, and (2382.0 ± 1309.0) μg/ml for L-D, M-D, and H-D REI groups, respectively], whereas the clearance, T_1/2_, and T_last_ of each group were identical. These results demonstrated that REI can be metabolized at a similar rate compared to EtOH and Ultravist®.

### Biosafety profiles of REI

Among the 16 rabbits selected for the safety evaluation experiment, 1 rabbit (6.3%) died after a high-dose EtOH injection. The venous and arterial blood of the animals were collected for hematologic examinations according to the time scale shown in Fig. 3d. During the 28 d observation period, alanine aminotransferase (ALT), aspartate aminotransferase (AST), alkaline phosphatase (ALP), serum creatinine, and urea levels have been within the normal range postoperatively in each group (*P* > 0.05; Fig. 3e; Additional file 1: Fig. S5). Additionally, REI endovascular administration did not cause remarkable changes to the animals’ ALT, AST, ALP, serum creatinine, blood urea, CKMB, HBDH, pO_2_, WBC, and HGB levels (*P* > 0.05; Fig. 3e; Additional file 1: Fig. S5). Histological examination of the rabbit organs after the observation period showed no pathological changes after REI injection (Additional file 1: Fig. S6).

### Clinical application of REI in VMs

**Fig. 4:**
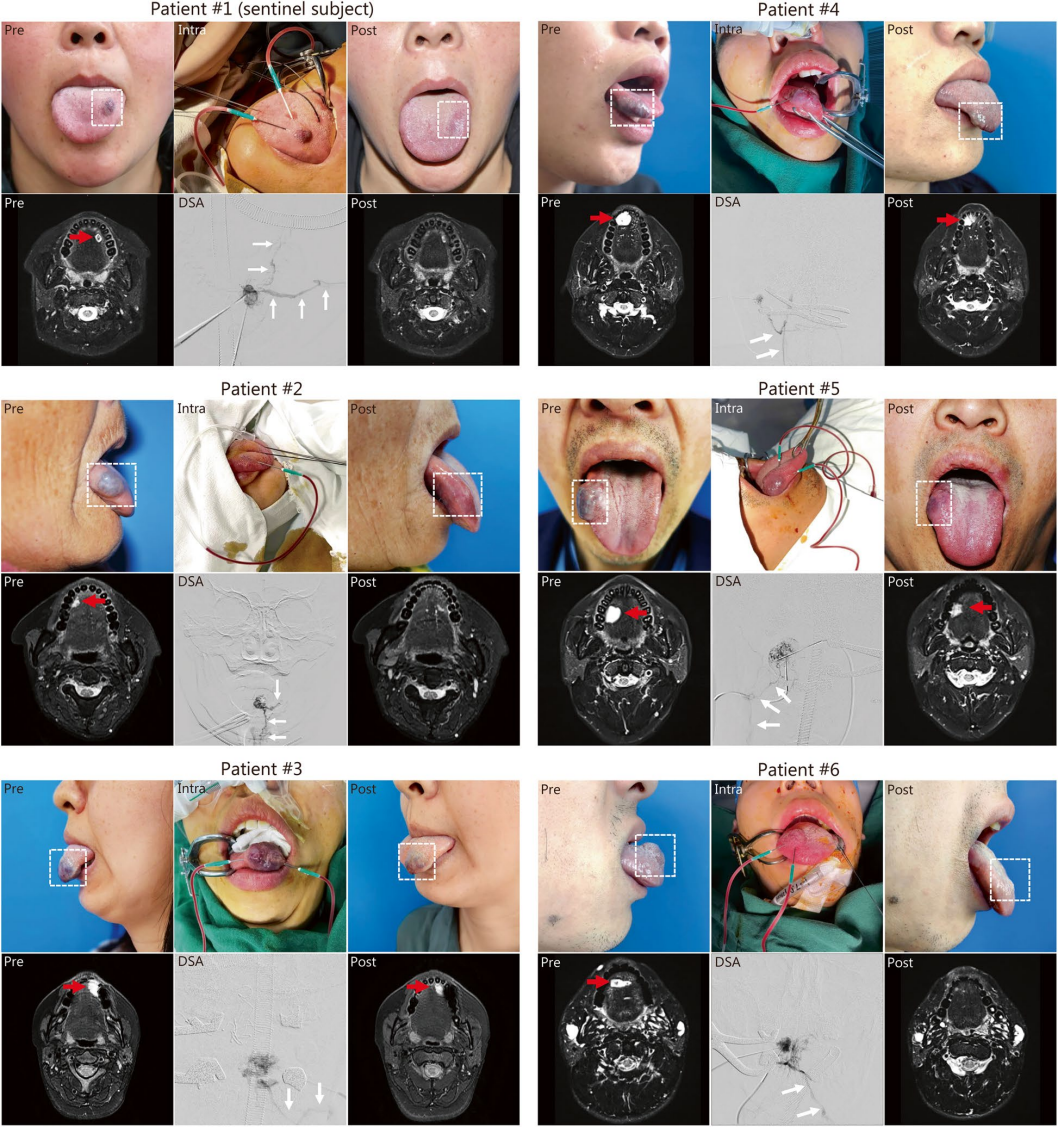
Sclerosis of patients with lingual venous malformations (VMs) by radiopaque ethanol injection (REI). Preoperative (Pre), intraoperative (Intra), and postoperative (Post) imaging and examinations of patient #1 (sentinel subject) and patients #2–6. Dotted rectangles indicate lesions of VMs; red arrows indicate VMs on magnetic resonance imaging; white arrows indicate outflowing veins of VMs. DSA digital subtraction angiography

Based on the promising effects of REIs in the pre-clinical experiments, an exploratory prospective study was performed to examine the safety of REI in clinical applications. The effectiveness of REIs in the treatment of peripheral VMs was also investigated. All patients were diagnosed with lingual VMs (blood reflux was noted through direct puncture, Fig. 4) and underwent one REI sclerotherapy procedure. No treatment-related adverse effects (AEs) or serious adverse effects (SAEs) occurred in the sentinel subject (Patient 1). Thereafter, we consecutively enrolled another 5 patients with lingual VMs, and neither AEs nor SAEs occurred during the safety observation period (Table 1).

**Table 1:** Clinical outcomes of single-time radiopaque ethanol injection (REI) sclerotherapy in patients with venous malformations (VMs)

Parameters	Patient 1^a^	Patient 2	Patient 3	Patient 4	Patient 5	Patient 6
Baseline data						
Age (years)	50	58	34	19	20	19
Sex (female/male)	Female	Female	Female	Male	Male	Male
Unifocal/multifocal	Unifocal	Unifocal	Unifocal	Unifocal	Unifocal	Unifocal
Previous treatments (no/yes)	No	No	No	No	No	No
Lesion volume (cm^3^)^b^	1.5	1.2	2.6	3.0	3.2	1.8
Treatment modalities						
Puig classification^c^	II	II	II	II	II	II
Dose of REI (ml)	2	1.5	4	5	5	3
Imaging under X-ray (absent/present)	Present	Present	Present	Present	Present	Present
Complications (no/yes)						
Cardiopulmonary accidents	No	No	No	No	No	No
Hepatic and renal functions	No	No	No	No	No	No
Swelling	Yes	Yes	Yes	Yes	Yes	Yes
Bleeding	No	No	No	No	No	No
Pain	No	No	No	No	No	No
Hemoglobinuria	No	No	No	No	No	No
Tongue dysfunction^d^	No	No	No	No	No	No
Local necrosis	No	No	No	No	No	No
Prognoses						
Residual lesion volume (cm^3^)^b^	NA	NA	1.1	1.3	1.0	0.2
Lesion reduction rate	100%	100%	58%	57%	77%	89%
Specialist efficacy score^e^	10	10	7	7	8	8
Overall efficacy	CR	CR	PR	PR	CR	CR

*NA* not available, *CR* complete response, *PR* partial response, *NR* no response

^a^The sentinel subject

^b^Measured by Mimics Medical 21.0 (Materialise, Leuven, Belgium)

^c^Puig classification of VMs: Type I: isolated lesion without peripheral drainage; Type II: lesion drains into normal veins; Type III: lesion drains into dysplastic veins; Type IV: venous ectasia

^d^Abnormal sensation or movement of the tongue indicating the injury of lingual nerve or hypoglossal nerve, respectively

^e^Evaluated by two independent specialists based on the Likert scale

All patients were treated with 20.5 ml REI [average: (3.4 ± 1.5) ml] and followed up for 2 months. In all 6 patients (100.0%), the REI successfully depicted the details of the VMs and was traced when draining into the outflowing veins under DSA (Fig. 4, white arrows). The lesions completely disappeared after REI sclerotherapy in 2/6 patients (33.3%). All patients (100.0%) scored > 6 in the specialist efficacy assessment. In total, 4/6 patients (66.7%) achieved complete response, and the remaining 2/6 patients (33.3%) showed partial response. Finally, all VM lesions were significantly reduced on magnetic resonance imaging (Fig. 4, red arrows). These results are preliminarily indicative of the promising efficacy of REI for the treatment of VMs.

## Discussion

In this study, iopromide was selected as an adjuvant to achieve radiopacity and improve the physical properties of ethanol, to compensate for current issues in ethanol interventional therapy. The compatibility between ethanol (81.4%, v/v) and iopromide (111.3 mg/ml) was optimized to produce REI with reliable stability. Animal experiments showed that REI had significant efficacy for venous sclerosis and arterial embolization while resulting in less tissue necrosis than EtOH. Pharmacokinetic analyses demonstrated that the ethanol and iopromide components of REI were easily metabolized by the body and did not induce acute or chronic injury to critical organs. Finally, sclerotherapy with REI in a cohort of patients with VMs confirmed the efficacy of REI.

EtOH is a well-known embolic agent and a sclerosant used to treat vascular malformations [21]. Ethanol embolization of arteriovenous fistulas (AVFs) was first reported by Sasaki et al. [22] in 1984. This innovative treatment had been popularized by Yakes et al. [23] in symptomatic vascular malformations (VMs, AVMs, congenital, and traumatic AVFs) since 1990. Percutaneous ethanol injection and transcatheter superselective ethanol embolization are critical methods for managing hepatic and renal tumors [24, 25]. However, the radiolucent nature of ethanol prevents novices from mastering ethanol interventional therapy [26]. Miyazono et al. [16] used iohexol powder to harvest an iohexol-ethanol solution that contained 72% ethanol by volume with 142 mgI/ml. Wysoki and White [27] reported that Visipaque (iodixanol, 320 mgI/ml) immediately crystallized after mixing with EtOH before VM sclerotherapy. They further tested several types of contrast media, including diatrizoate meglumine, gadodiamide, iohexol, iothalamate meglumine, diatrizoate meglumine, and iodixanol, but they all precipitated with ethanol.

In addition to iodixanol, both iomeprol and ioversol also showed poor compatibility with ethanol in a pilot study (data not shown). Finally, iopromide, a low-permeability nonionic contrast agent [28], was screened as a candidate. Iopromide is easily soluble in water but only slightly soluble in ethanol [18]. We detected the Z1, Z2, E1, and E2-configurations of iopromide in the REI samples (Fig. 1c). During our observation, the proportion between different configurations directly led to the overall stability of the solution, and the Z-configuration (maintaining the stability of the solution) gradually converted into the E-configuration (leading to crystallization and precipitation) after mixing with ethanol. This configuration transformation did not stop until the REI solution reached equilibrium.

Denaturation of proteins under the action of EtOH is caused by the breakage of hydrogen bonds between protein molecules and the disintegration and gelation of the three-dimensional structure of the protein [29]. This denaturation, which occurs within seconds, is irreversible and leads to the breakdown of all red blood cells, vascular endothelial cells, and perivascular tissues upon contact with ethanol. When the ethanol concentration is < 70%, a reversible reaction occurs [14]. EtOH exerts the most potent efficacy at each concentration of ethanol; however, another main drawback of using EtOH is the high risk of complications, such as superficial ulcerative necrosis of the extremities, especially the peripheral appendages [30]. Kim et al. [17] suggested that an ethanol concentration of 80–100% was considered therapeutic. However, there is a lack of systematic in vivo experiments and prospective clinical studies to determine whether appropriately reducing the concentration of ethanol could preserve therapeutic capacity and improve safety.

Our results also highlight the significance of the elevated viscosity of the ethanol injection. The contact time with the blood vessel wall directly affects the efficiency of ethanol. To treat vascular malformations, especially high-flow malformations (AVMs and AVFs), mechanical embolic agents (coils or occluders) are first used to curb the flow rate and provide favorable conditions for ethanol [31]. Hu et al. [32] reported that the high diffusivity of EtOH limited its therapeutic window, although the raw material was cost-effective and easy to access. Legiehn [33] suggested that ethanol could be mixed with various contrast water or lipid-soluble agents to increase viscosity. The REI solution had three times more viscosity than EtOH, which further prolonged the time of ethanol in the lumen. Among previous products, Ethibloc®, which consists of maize protein zein, sodium diatrizoate, EtOH, and Oleum papaveris, is a viscous, radiopaque emulsion. Zein is insoluble in water and precipitates immediately upon contact with the blood. Thus, when injected intravascularly, Ethibloc® hardens immediately and completely occludes the vessel [34]. Its principle of vascular occlusion is similar to that of Onyx®, that is, involving mechanical embolization [32].

However, Ethibloc® does not achieve satisfactory outcomes in peripheral vascular malformations, and thus, its use has gradually decreased in clinical practice [35]. Aside from Ethibloc®, lipiodol is commonly blended with EtOH to enhance radiopacity and viscosity in managing renal and hepatic cancers [15, 36, 37]. However, an ethanol-lipiodol mixture is a kind of emulsion, containing two immiscible liquid phases, where the dispersed phase is present as globules and the continuous phase is the other liquid [38]. An ethanol-lipiodol emulsion has an unstable nature, and a two-phase separation can easily occur when this heterogeneous system integrates into the blood flow, not alone in high-flow vascular malformations, preventing real-time monitoring of ethanol reflux. Therefore, this method has seldom been applied to vascular malformations [39]. Additionally, Sannier et al. [12] reported the use of ethylcellulose-ethanol (gelified ethanol), which has better viscosity than EtOH, in VM treatment. In addition, the prompt precipitation of ethylcellulose when in contact with blood was expected to lead to expansion during injection. The same authors mixed gelified ethanol with lipiodol to achieve radiopacity [40], but no related clinical trials or mature products have been reported since then. In our prospective pilot trial on lingual VMs, only one REI procedure achieved satisfactory outcomes with minor complications.

Finally, although transgenic mouse models of VMs and AVMs have been established through conditional mutation of *Pik3ca* [41], *Kras* [42], *Endoglin* [43], and *Alk1* [44], the murine lumen is too narrow to support interventional operations. The current study tested the efficacy of REI in three animal models. The rabbit ear has a clearly distributed vasculature and a simple structure suitable for observation and is considered a competent area for venous sclerosis or arterial embolization [45]. Moreover, auricular vascular occlusion does not significantly affect longevity, providing favorable conditions for survival [46]. Given the different presentation of venous and arterial occlusions, we developed two comprehensive evaluation systems to assess effectiveness from the perspective of appearance, angiography, and histopathology, as well as from macro- to micro-dimensions.

As the outflowing canal, sclerosis of the simple auricular vein would not cause diffused occlusion of the vasculature and extensive necrosis of tissue; therefore, we focused on the pathological changes at the injection site. REI completely occluded the auricular vein, similar to EtOH, but led to less tissue necrosis, even at a higher dose per bolus than EtOH (Fig. 2d, Additional file 1: Table S5). In contrast to vein embolization, auricular arterial embolization removed the tissue blood supply from the injection point. Tissue necrosis caused by arterial ethanol embolization resulted in dry gangrene of the distal tissue during long-term observation (Additional file 1: Fig. S2a). Interestingly, REI resulted in broader occlusion of the auricular vasculature (Additional file 1: Fig. S2b), but it also caused more concentrated tissue necrosis than EtOH (Additional file 1: Fig. S2c). These results provide evidence for the embolization capacity of REI in parenchymatous organs (Additional file 1: Fig. S3), further highlighting the possibilities for the application of TAE.

This study has some limitations. First, vascular malformations’ vasculature is complex and is endowed with various gene mutations; however, we carried out the pre-clinical experiments on normal vessels with a limited sample size. Next, the indication of REI was selected as VMs in the present work; expanding the indication to AVMs should be carried out in the future. Finally, the group size of a clinical trial was relatively small in the present research. Given its exploratory nature, we could only obtain the preliminary results of REI’s clinical application. Nevertheless, a clinical trial on simple VMs with a larger sample size will be carried out in future research.

## Conclusions

The present study introduced a novel REI based on iopromide to compensate for the radiolucency of EtOH in the treatment of vascular malformations. The REI had a higher viscosity than EtOH, further increasing the actuation duration with the vascular wall. Pre-clinical animal experiments supported the efficacy of REI in venous sclerosis, arterial embolization, and TAE of the kidney. Furthermore, REI can be metabolized efficiently by body, and presents biosafety in a therapeutic dose. The promising results of REI in the sclerotherapy of VMs indicated its potential for clinical application.

### Supplementary Information

**Additional file 1: Materials and methods.** **Fig. S1** In vivo experimental methods of rabbits. **Fig. S2** Embolization of central auricular artery by radiopaque ethanol injection (REI). **Fig. S3** Transcatheter arterial embolization (TAE) of the kidney by radiopaque ethanol injection (REI). **Fig. S4** Transcatheter arterial embolization (TAE) of renal artery by radiopaque ethanol injection (REI). **Fig. S5** Supplementary indices of hepatic, renal and cardiac functions. **Fig. S6** Histopathological staining of rabbits’ pivotal organs. **Table S1** The scoring criteria of auricular venous histopathology. **Table S2** The scoring criteria of auricular appearance. **Table S3** The scoring criteria of auricular angiography. **Table S4** The scoring criteria of auricular arterial histopathology. **Table S5** Angiography of auricular vein and necrosis of auricular tissue [*n*(%), *n* = 6]. **Table S6** Pharmacokinetic parameters of ethanol in rabbits of each group. **Table S7** Pharmacokinetic parameters of iopromide in rabbits of each group (mean ± SD).

## Data Availability

All data generated or analyzed during this study are included in this published article and its supplementary information files.

## Abbreviations

AVMs: Arteriovenous malformations
AVFs: Arteriovenous fistulas
AUC: Area under the curve
C_max_: Maximum concentration;
DSA: Digital subtraction angiography
EtOH: Absolute ethanol
MRT: Mean residence time
REI: Radiopaque ethanol injection
T_1/2_: Elimination half-life
TAE: Transcatheter arterial embolization
TEM: Transmission electron microscopy
Vdss: Volume of distribution at steady state
T_last_: Time of last observed concentration
VMs: Venous malformations
WFI: Water for injection
