# Supplementary material for: Novel radiopaque ethanol injection: physicochemical properties, animal experiments, and clinical application in vascular malformations
Source: Mil Med Res. 2024 Jun 20;11:39. doi: 10.1186/s40779-024-00542-7 (PMC11188249; doi:10.1186/s40779-024-00542-7)
Supplement: Supplementary file 1 — Additional file 1: Materials and methods. Fig. S1 In vivo experimental methods of rabbits. Fig. S2 Embolization of central auricular artery by radiopaque ethanol injection (REI). Fig. S3 Transcatheter arterial embolization (TAE) of the kidney by radiopaque ethanol injection (REI). Fig. S4 Transcatheter arterial embolization (TAE) of renal artery by radiopaque ethanol injection (REI). Fig. S5 Supplementary indices of hepatic, renal and cardiac functions. Fig. S6 Histopathological staining of rabbits’ pivotal organs. Table S1 The scoring criteria of auricular venous histopathology. Table S2 The scoring criteria of auricular appearance. Table S3 The scoring criteria of auricular angiography. Table S4 The scoring criteria of auricular arterial histopathology. Table S5 Angiography of auricular vein and necrosis of auricular tissue [n(%), n = 6]. Table S6 Pharmacokinetic parameters of ethanol in rabbits of each group. Table S7 Pharmacokinetic parameters of iopromide in rabbits of each group (mean ± SD). [file 40779_2024_542_MOESM1_ESM.pdf]

Materials and methods

Viscosity measurement

The viscosities of absolute ethanol (EtOH) and the radiopaque ethanol injection (REI) solution were measured using a viscometer (DV3T; AMETEK, Brookfield, MA, USA). Briefly, we set the temperature to 25 °C and 37 °C and measured the sample using rotor #18 at a speed of 250 r/min.

Purity testing

The purity of the REI solution was determined using high-performance liquid chromatography (HPLC) (LC-2030C, Shimadzu, Kyoto, Japan). For the test solution, an appropriate amount of REI was measured and diluted with water-acetonitrile to produce a solution containing 2 mg iopromide per milliliter. For the control solution, an appropriate amount of REI was measured and diluted it with water-acetonitrile to produce a solution containing 10 μg/ml iopromide. The chromatographic conditions were as follows: octadecyl silane-bonded silica gel was used as the filler; monosodium phosphate solution-acetonitrile (97:3) as mobile phase A, and acetonitrile as mobile phase B. Gradient elution was then conducted as follows. Time (0 min): phase A (100%), phase B (0%); Time (40 min): phase A (100%), phase B (0%); Time (80 min): phase A (85%), phase B (15%); Time (95 min): phase A (25%), phase B (75%); Time (100 min): phase A (100%), phase B (0%); Time (110 min): phase A (100%), phase B (0%). The detection wavelength was 242 nm. For the determination method, test and control solutions were injected into the HPLC system, and chromatograms were recorded. If impurity peaks were present in the test solution chromatogram, the impurity content was calculated using the principal component self-comparison method.

Ethanol content detection of REI

The ethanol content of the REI solution was determined using high-performance gas chromatography (HPGC; 7890 B, Agilent, CA, USA). For the test solution, we measured 1 ml of the REI and diluted it with water to 50 ml and then measured 0.8 ml of this solution and diluted it to 20 ml. For the control solution, 200 mg EtOH (Brilliant Pharmaceutical Co., Ltd., Chengdu, China) was diluted with water to 20 ml; then, 1.0 ml of this solution was diluted with water to 20 ml. The chromatographic conditions

were as follows: a capillary column with 6% cyanopropylphenyl and 94% dimethylpolysiloxane as the stationary liquid was used as the chromatographic column; the initial temperature was set as 40 ℃; and a programmed temperature chromatography was performed. Nitrogen was used as the carrier gas, and a flame ionization detector was used. Next, we separately injected the test and control solutions into the high-performance gas chromatography (HPGC) system and recorded chromatograms. An external standard was used to calculate peak areas.

Iopromide content detection of REI

The iopromide content of the REI was detected using HPLC (LC-20AD, Shimadzu, Kyoto, Japan). For the test solution, the appropriate amount of REI was diluted with methanol-water to produce a solution containing 1.6 mg of iopromide per milliliter. For the control solution, the appropriate amount of iopromide (Brilliant Pharmaceutical Co., Ltd., Chengdu, China) was diluted with methanol-water to produce a solution containing 1.6 mg of iopromide per milliliter. The chromatographic conditions were as follows: octadecyl-silane-bonded silica gel was selected as the filler and chloroform-methanol-water (4:75:900) was used as the mobile phase. Gradient elution was conducted, and the detection wavelength was 254 nm. For the determination method, test and control solutions were injected into the HPLC system, and chromatograms were recorded. The external standard method was used to calculate the sum of the peak areas.

Quantification of plasmatic ethanol and iopromide

The plasma iopromide concentration was quantitatively analyzed using liquid chromatography-tandem mass spectrometry (LC-MS/MS; LC-40D, Shimadzu, Kyoto, Japan; Triple Quad 6500^{+}, AB SCIEX, USA). The linear range was 0.1000 – 200.0 μg/ml, and the lower limit of quantification (LLOQ) was 0.1000 μg/ml. The concentrations of quality control (QC) samples were 0.3000 (QCD), 8.000 (QCC), 80.00 (QCB), and 160.0 μg/ml (QCA).

The concentration of plasma ethanol was quantitatively analyzed using gas chromatography-triple quadrupole tandem mass spectrometry (GC-MS/MS; 8890GC; 7000D GC/TQ, Agilent, USA). In the first standard curve, the linear range was 0.5000 – 50.00 μg/ml; the LLOQ was 0.5000 μg/ml; and the concentration of QC samples was 1.5000 μg/ml (LQC), 18.00 μg/ml (MQC), and 40.00 μg/ml (HQC).

In the second standard curve, the linear range was 0.2500 – 20.00 μg/ml; the LLOQ was 0.5000 μg/ml; and the concentrations of QC samples were 0.7500 μg/ml (LQC), 8.000 μg/ml (MQC), and 16.00 μg/ml (HQC). In the third standard curve, the linear range was 10.00 – 1000 μg/ml; the LLOQ was 10.00 μg/ml; and the concentrations of QC samples were 30.00 μg/ml (LQC), 400.0 μg/ml (MQC), and 750.0 μg/ml (HQC). Values below the LLOQ were reported as 0 ng/ml.

Inclusion and exclusion criteria

Inclusion criteria

Patients who met the following criteria were included: voluntarily participated in the clinical trial, signed the informed consent form, understood, and followed the study procedures; aged 18 – 65 years, regardless of sex; body mass index (BMI) [BMI = weight (kg)/height^2 (m^2)] within the range of 18.5 – 28.0 kg/m^2 (including boundary values); venous malformations (VMs) diagnosed by magnetic resonance imaging (MRI) with the following conditions: (1) common VMs of the tongue (ISSVA, 2018 [1]); (2) single lesion which could be measured under MRI (the shortest diameter of the lesion was ≥ 10 mm); (3) the contour of lesion was clear, and the maximum diameter of lesion was ≤ 5 cm; (4) the lesion was not suitable for observation or conservative treatment or the symptoms worsened after observation or conservative treatment, indicating the need for sclerotherapy; (5) no complications such as ulceration, infection, or bleeding had not occurred in the lesion; no previous treatment for VMs, including, but not limited to, surgical therapy, sclerotherapy, laser therapy, cryoablation, radiofrequency ablation, and targeted therapy (e.g., rapamycin), except for symptomatic therapy (e.g., analgesia and anticoagulation), was received within 3 months prior to treatment; adequate organ functions, including (1) normal all routine blood examination parameters; (2) normal levels of aspartate aminotransferase (AST), alanine aminotransferase (ALT), and total bilirubin; baseline level of albumin ≥ 30 g/L; (3) creatinine clearance ≥ 50 ml/min.

Exclusion criteria

Patients who met the following criteria were excluded: any other type of vascular malformations, such as capillary malformations, arteriovenous malformations, and lymphatic malformations; VMs involving the gastrointestinal tract, muscles, bones, joints, or nervous system; mixed or complex VMs

or VMs with other lesions (e.g., capillary-venous malformations, lymphatic-venous malformations, capillary-lymphatic-venous malformations, capillary-venous arteriovenous malformations, capillary-lymphatic-venous arteriovenous malformations, verrucous venous malformations, familial cutaneous mucous VMs, blue rubber bleb nevus syndrome, glomuvenous malformation, Klippel-Trenaunay syndrome, and Maffucci syndrome); deep VMs whose lesions could not be reached by a percutaneous puncture; presence of intracranial VMs in the communicating branches of the vessels; the VMs lesions were located at the base of the tongue, floor of the mouth, pharynx, larynx, parapharyngeal space, soft palate, or neck. To maintain the patency of the upper respiratory tract, it was estimated that prophylactic tracheotomy was required before surgery, emergency tracheotomy was needed during surgery, or tracheal intubation was reserved for ≥ 48 h after surgery; had allergic history of iodine contrast media or ethanol; experienced active infections as follows: (1) active hepatitis B virus (HBV) or hepatitis C virus (HCV) infection; (2) history of human immunodeficiency virus (HIV) infection or acquired immunodeficiency syndrome (AIDS); (3) treponema pallidum antibody (TP-Ab) positive (TP-Ab positive patients confirmed negative by rapid plasma reagent test or toluidine red unheated serum test were enrolled); (4) other active infections, such as acute pneumonia and unexplained persistent fever, requiring intravenous anti-infective therapy 7 d before enrollment; had severe systemic diseases that make it difficult to tolerate general anesthesia, including but not limited to: (1) congestive heart failure (New York Heart Association grade III/IV heart failure [2]) or left ventricular ejection fraction < 50%; (2) myocardial infarction, severe or unstable angina pectoris, stroke, pulmonary embolism, arterial thrombosis, and deep vein thrombosis occurred within 6 months before enrollment; (3) pulmonary hypertension or right-to-left shunt; (4) uncontrolled ventricular arrhythmia; (5) hypertension not well controlled with antihypertensive medications (systolic blood pressure ≥ 160 mmHg and diastolic blood pressure ≥ 110 mmHg); (6) uncontrolled diabetes, thyroid disease, and other endocrine disorders; (7) albuminuria ≥ (++) was further measured at 24 h of total urinary protein > 1.0 g; (8) abnormal coagulation function [any of these abnormalities: prothrombin time >3 s longer than upper limit of normal (ULN), activated partial thrombin time > 10 s longer than ULN, international normalized ratio > 2.0, fibrinogen less than lower limit of normal], bleeding tendency, receiving thrombolytic therapy or anticoagulation treatment, or a diagnosis of localized intravascular coagulation; the serum pregnancy test of fertile female subjects was positive within 3 d before enrollment; fertile women and men with

fertile partners were unable to use adequate barrier contraceptive methods for contraception or to avoid sex during the study period; major surgery or other organ intervention and ablation within 28 d before enrollment; a seasonal influenza vaccine that did not contain the live virus was allowed if the patient had been vaccinated with the live or attenuated vaccine within 28 d of enrollment; participation in other clinical trials within 3 months before screening (based on the time of last use of the study drug); history of a malignant tumor within 5 years before enrollment; any other medical, mental, or social condition that the investigator considered likely to impair the subject's rights, safety, welfare, or ability to sign an informed consent form and cooperate and participate in the study, or that would interfere with the interpretation of results.

Protocol of REI sclerotherapy

All operative procedures were performed under general anesthesia. All patients received an intravenous injection of dexamethasone 10 min preoperatively to control inflammation and concurrent swelling. Electrocardiography was performed throughout the operation, and invasive arterial pressure monitoring was performed by catheterization of the patient's radial artery.

Sclerotherapy of VMs using REI was performed by an interventional radiologist with at least 5 years of experience. Based on our previous report [3], a 21G butterfly needle was used for direct puncture. Once venous blood was observed, REI was injected to perform dynamic venography based on digital subtraction angiography. The location of the needle tip, extent of the lesion, and condition of the drainage vein were detected using venography. Simultaneously, the Puig classification [4] of VMs was evaluated. Patients with types I and II underwent a single procedure of REI sclerotherapy. The total dose per procedure was approximately 10 ml. The pressure and speed of injection were adjusted based on real-time imaging. Multipoint puncture was performed, as needed, for adequate sclerotherapy in a single procedure.

All patients underwent postoperative fluid replacement. Omeprazole was used to reduce the risk of gastrointestinal stress, ketorolac tromethamine was used as an analgesic, and sodium aescinate was used for detumescence. Before discharge, the clinical outcomes including signs, symptoms, and complications were recorded.

Evaluation of safety and effectiveness

Safety

Safety indicators included monitoring of vital signs (e.g., body temperature, pulse, heart rate, respiration, blood oxygen saturation, and blood pressure), physical examination results, and laboratory examination results (e.g., blood routine, biochemical assay, urine routine, and coagulation functions) during the treatment period. Adverse events (AEs) and serious adverse events (SAEs) were evaluated according to the National Cancer Institute Common Terminology Criteria for Adverse Events (NCI-CTCAE) version 5.

Effectiveness

The efficacy of REI sclerotherapy was evaluated from two aspects: the traceability of the REI under DSA throughout the entire injection and the therapeutic effect 2 months after a single injection, including the following indicators: (1) Treatment effectiveness. Two independent radiologists evaluated and measured the changes in lesion volume before and after treatment using MRI. The lesion volume was examined by MRI examination (Magnetom Aera, Simens, Germany; MR750, Discovery™, GE, USA) using 3-mm-thick slices. The image data of each patient were exported in Digital Imaging and Communications in Medicine (DICOM) format. The abnormal area of the fat-suppressed T_2 weighted sequence was reconstructed using Mimics Medical 21.0 (Materialize, Leuven, Belgium). Thereafter, the volume of the VM lesions was determined. (2) Specialist efficacy score. Two independent specialists collected detailed medical histories and performed physical examinations of the subjects before and after treatment. Images of the lesions before and after treatment were also compared. The effect of treatment on clinical symptoms and physical examination were both evaluated and scored using a Likert scale (very satisfied = 5 points, satisfied = 4 points, neutral = 3 points, dissatisfied = 2 points, and very dissatisfied = 1 point). The sum of the two scores was the specialist's score. (3) Overall efficacy. Treatment response was classified as complete response (i.e., > 75% reduction in lesion volume as evaluated by MRI and a specialist efficacy score ≥ 8), partial response (i.e., 50 – 75% reduction in lesion volume as assessed by MRI or specialist efficacy score > 6), and no response (i.e., < 50% reduction in lesion volume as assessed by MRI or specialist efficacy score ≤ 6).

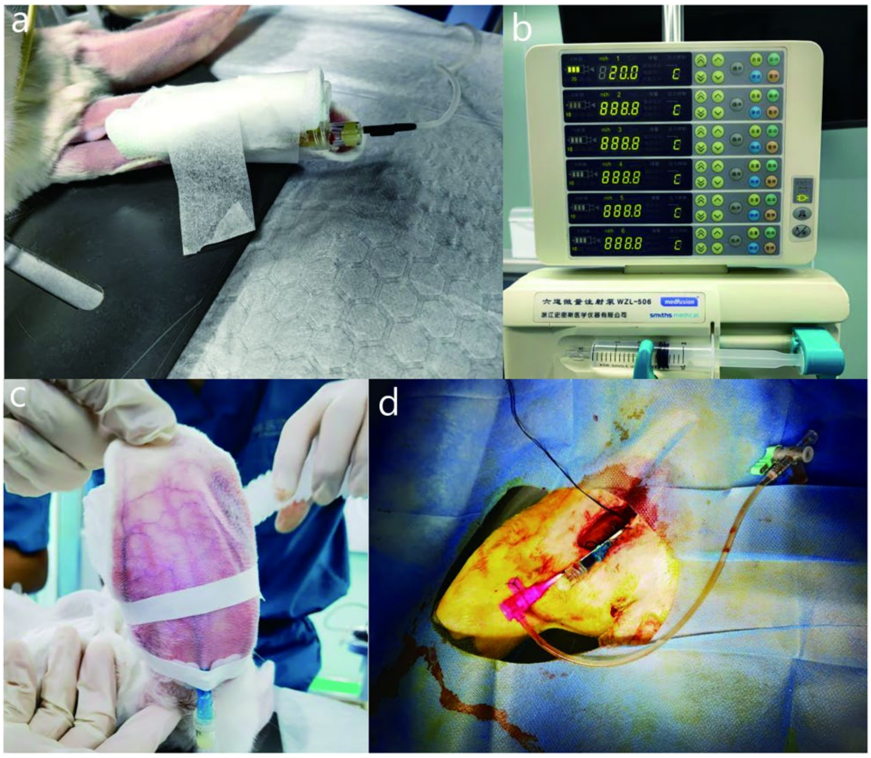

Fig. S1 In vivo experimental methods of rabbits. **a** Catheterization of auricular vein. **b** Parameters of micropump. **c** Catheterization of central auricular artery. **d** Catheterization of right femoral artery.

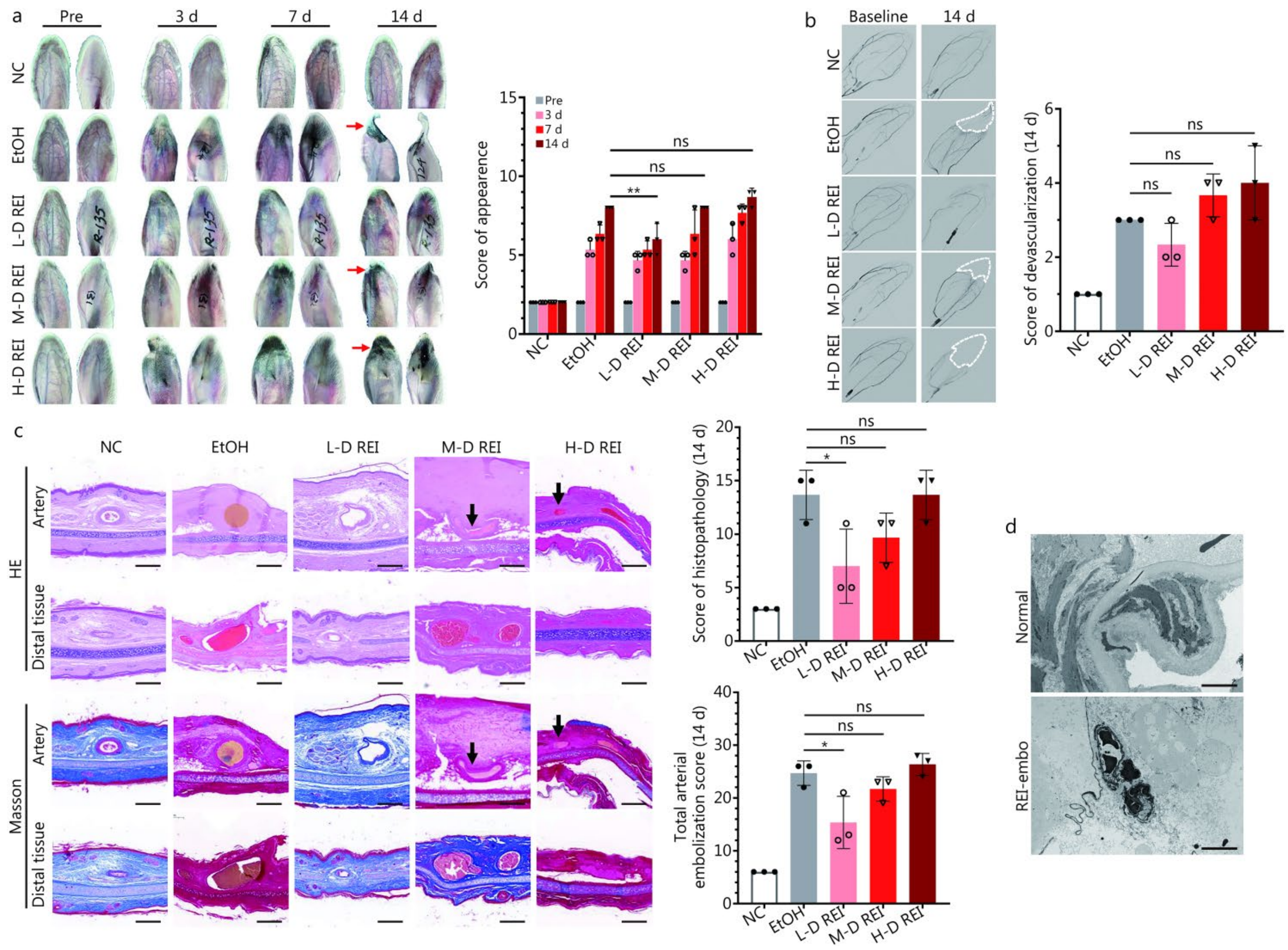

Fig. S2 Embolization of central auricular artery by radiopaque ethanol injection (REI). **a** Dynamic change in the general appearance of the ear after arterial embolization, and appearance score by group (14 d: $P < 0.0001$, tested by one-way ANOVA). Red arrows indicate the necrosis of auricular distal tissue. **b** Baseline and 14-d postoperative angiography of the auricular artery, and devascularization score by group ($P = 0.0006$, tested by one-way ANOVA). **c** Cross-sectional HE and Masson staining of embolic site of the artery and distal tissue of the ear. Black arrows indicate the formation of thrombus in the lumen. Scale bar = 0.5 mm. Histopathology score by group ($P = 0.0011$, tested by one-way ANOVA) and total arterial embolization score by group ($P < 0.0001$, tested by one-way ANOVA) were calculated. **d** Transmission electron microscopy (TEM) of the arterial lumen [normal artery and artery with REI embolization (REI-embo)]. Scale bar = 5 μm. $^{*}P < 0.05$, $^{**}P < 0.01$ (Tukey test of post-hoc analysis). NC: 0.250 ml/kg saline; EtOH: 0.250 ml/kg EtOH; L-D REI: 0.125 ml/kg REI; M-D REI: 0.250 ml/kg REI; H-D REI: 0.375 ml/kg REI ($n = 3$). ns not significant, EtOH absolute ethanol, NC negative control, L-D low-dose, M-D moderate-dose, H-D high-dose, Pre preoperative

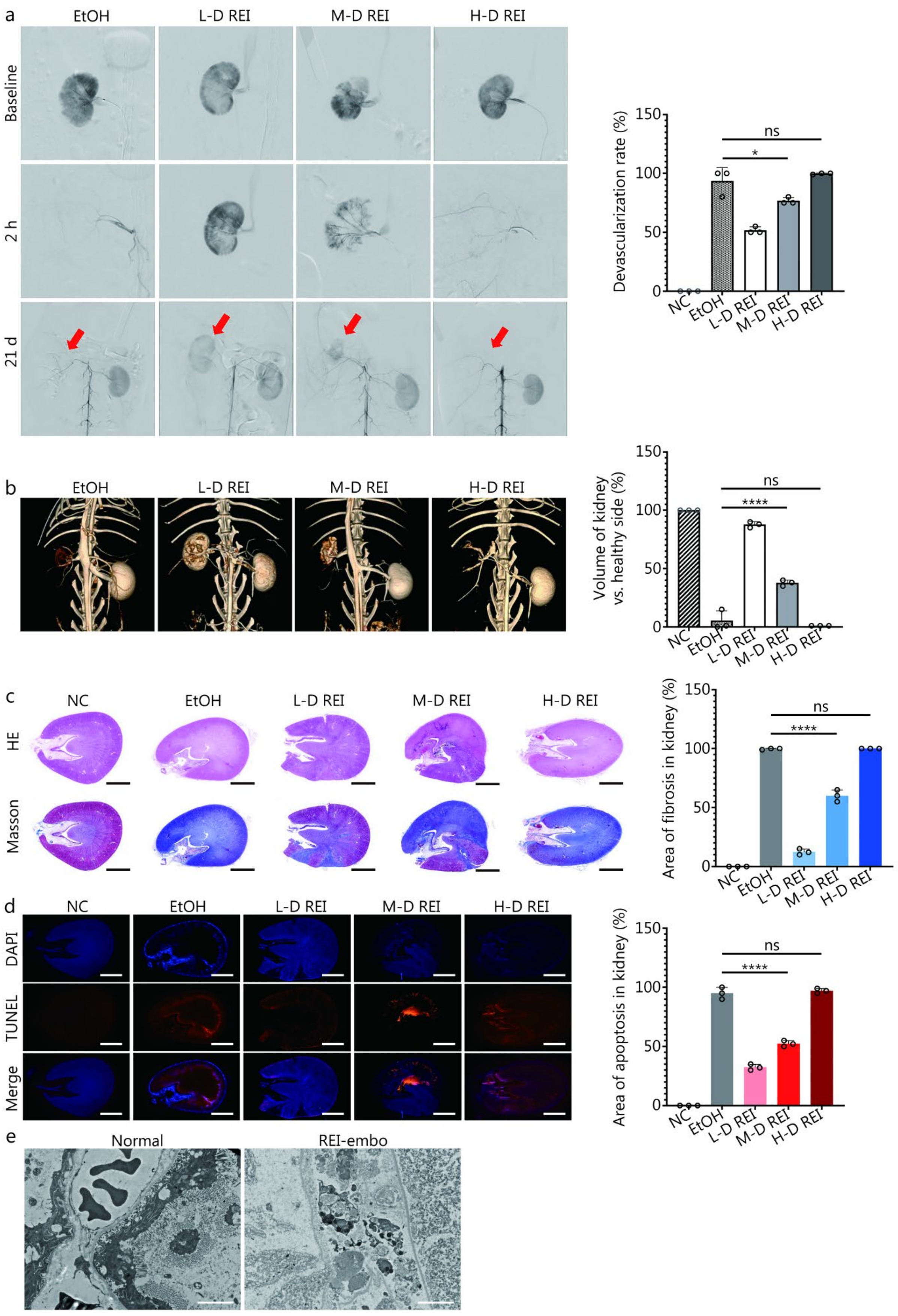
a
EtOH
L-D REI
M-D REI
H-D REI
Baseline
2 h
21 d
Devascularization rate (%)
ns
*
NC
EtOH
L-D REI
M-D REI
H-D REI
b
Volume of kidney vs. healthy side (%)
****
c
HE
Masson
Area of fibrosis in kidney (%)
d
DAPI
TUNEL
Merge
Area of apoptosis in kidney (%)
e
Normal
REI-embo

Fig. S3 Transcatheter arterial embolization (TAE) of the kidney by radiopaque ethanol injection (REI). **a** Representative renal angiography image in each group, and devascularization rate in renal angiography 21 d after embolization ($P < 0.0001$, tested by one-way ANOVA). Red arrows indicate the renal angiography of embolized side. **b** Representative three-dimensional reconstruction of the kidney, and ratio of embolic renal volume to healthy renal volume 21 d after embolization ($P < 0.0001$, tested by one-way ANOVA). **c** Cross-sectional HE and Masson staining of the kidney 21 d after embolization, and quantitative analysis of renal fibrotic area ($P < 0.0001$, tested by one-way ANOVA). Scale bar = 5 mm. **d** Cross-sectional TUNEL immunofluorescence of the kidney 21 d after embolization, and quantitative analysis of renal apoptotic area ($P < 0.0001$, tested by one-way ANOVA). Scale bar = 5 mm. **e** Transmission electron microscopy (TEM) images of kidney tissue. Scale bar = 5 μm. $^{*}P < 0.05$, $^{****}P < 0.0001$ (Tukey test of post-hoc analysis). NC: 0.250 ml/kg saline; EtOH: 0.250 ml/kg EtOH; L-D REI: 0.125 ml/kg REI; M-D REI: 0.250 ml/kg REI; H-D REI: 0.375 ml/kg REI (n = 3). ns not significant, REI-embo REI embolization, EtOH absolute ethanol, NC negative control, L-D low-dose, M-D moderate-dose, H-D high-dose

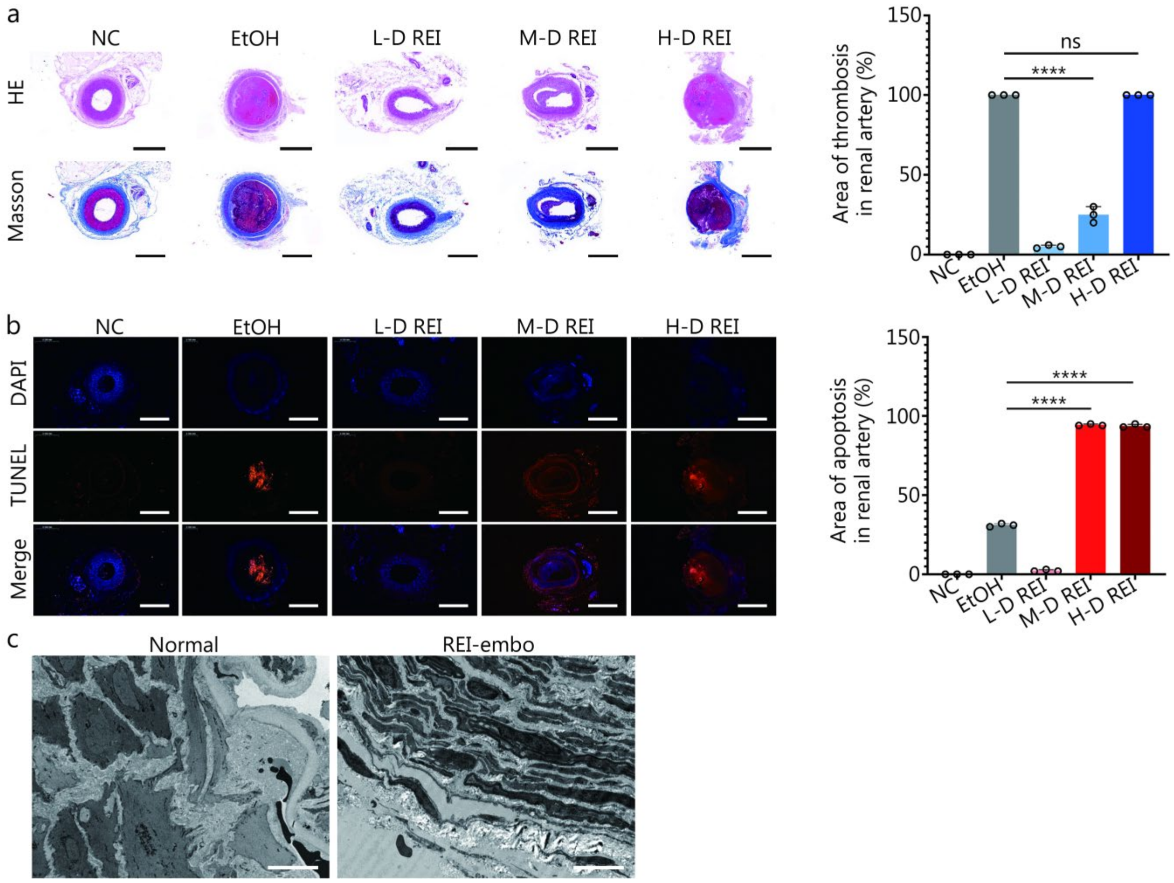

Fig. S4 Transcatheter arterial embolization (TAE) of renal artery by radiopaque ethanol injection (REI). **a** Cross-sectional HE and Masson staining of kidney 21 d after embolization, and quantitative analysis of thrombotic area in renal artery ($P < 0.0001$, tested by one-way ANOVA). Scale bar = 5 mm. **b** Cross-sectional TUNEL immunofluorescence of kidney 21 d after embolization, and quantitative analysis of apoptotic area in renal artery ($P < 0.0001$, tested by one-way ANOVA). Scale bar = 5 mm. **c** Transmission electron microscopy (TEM) of renal arterial lumen [normal artery and artery with REI embolization (REI-embo)]. Scale bar = 10 μm. $^{****}P < 0.0001$ (Tukey test of post-hoc analysis). NC: 0.250 ml/kg of saline; EtOH: 0.250 ml/kg of absolute ethanol; L-D REI: 0.125 ml/kg of REI; M-D REI: 0.250 ml/kg of REI; H-D REI: 0.375 ml/kg of REI (n = 6). ns not significant, EtOH absolute ethanol, NC negative control, L-D low-dose, M-D moderate-dose, H-D high-dose

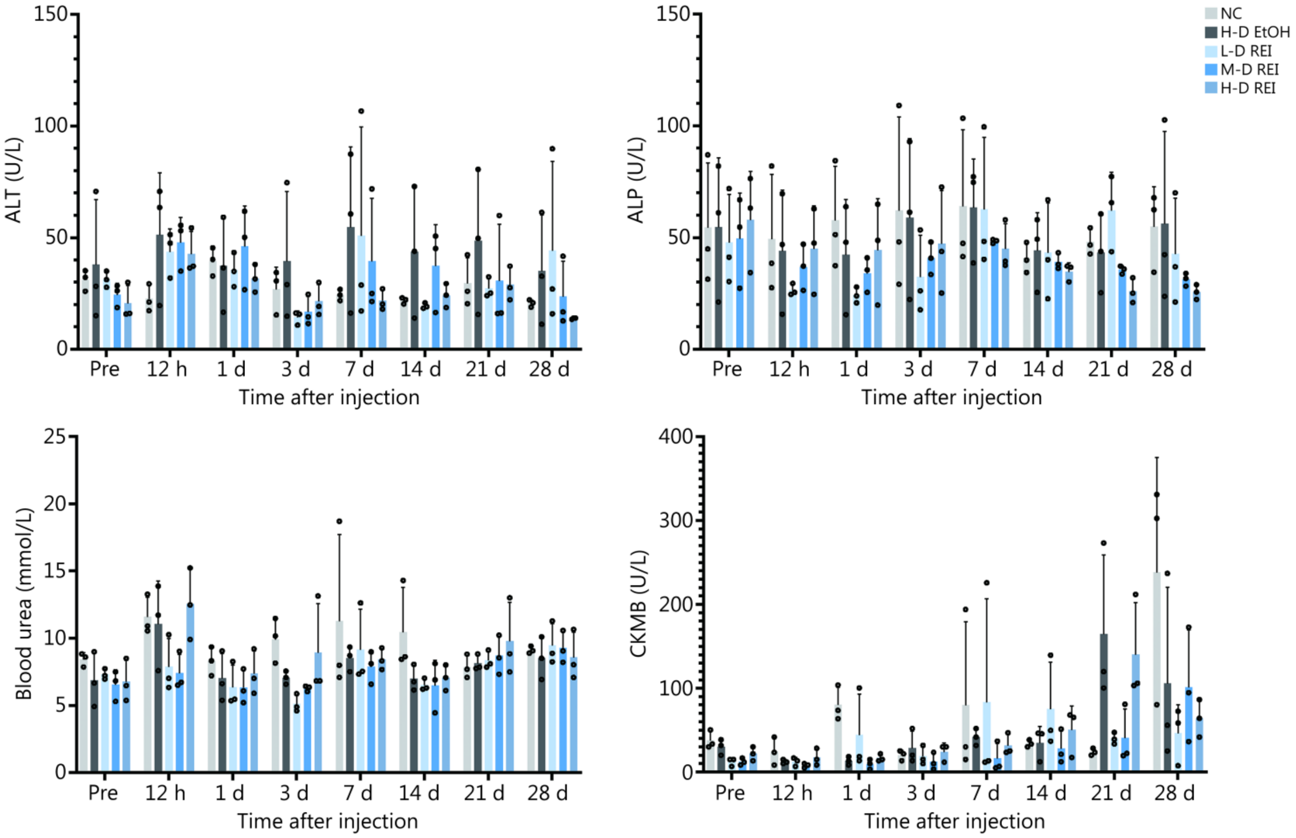

Fig. S5 Supplementary indices of hepatic, renal and cardiac functions. Dynamic changes of aspartate transaminase (AST), alkaline phosphatase (ALP), blood urea, and creatine kinase-MB (CKMB) levels of animals ($P > 0.05$, tested by one-way ANOVA at each time point). NC: 0.250 ml/kg of saline; H-D EtOH: 0.375 ml/kg of absolute ethanol; L-D REI: 0.125 ml/kg of REI; M-D REI: 0.250 ml/kg of REI; H-D REI: 0.375 ml/kg of REI. EtOH absolute ethanol, NC negative control, L-D low-dose, M-D moderate-dose, H-D high-dose, REI radiopaque ethanol injection

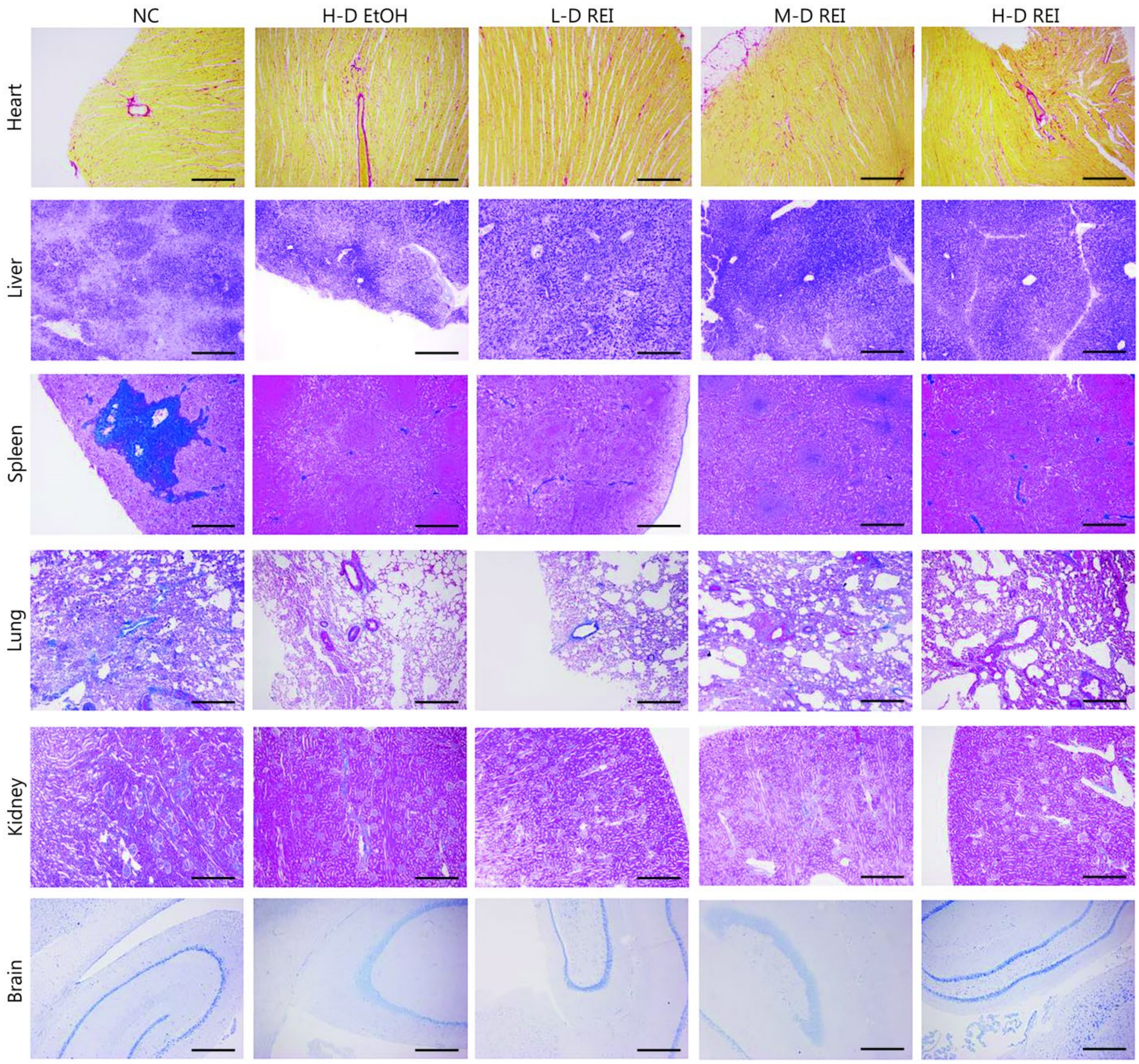

Fig. S6 Histopathological staining of rabbits' pivotal organs. The heart tissue was stained by Sirius red; liver tissue was stained by periodic acid Schiff; spleen, lung, and kidney tissue were stained by Masson staining; brain tissue was stained by toluidine blue. Scale bar = 1 mm. NC: 0.250 ml/kg of saline; H-D EtOH: 0.375 ml/kg of absolute ethanol; L-D REI: 0.125 ml/kg of REI; M-D REI: 0.250 ml/kg of REI; H-D REI: 0.375 ml/kg of REI. EtOH absolute ethanol, NC negative control, L-D low-dose, M-D moderate-dose, H-D high-dose, REI radiopaque ethanol injection

Table S1 The scoring criteria of auricular venous histopathology

**Grade of histopathology**	**Score**
Occlusion of auricular vein	
None	1
Partial	3
Complete	5
Fibrosis of auricular vein	
No fibrosis	1
Fibroblast formed and inflammatory cell infiltrated	2
Formation of granulation	3
Formation of extensive fibrocyte	4
Complete fibrosis	5
Damage of perivascular tissue	
No damage	1
Infiltration of inflammatory cells	2
Tissue thickening with proliferation of granulation	3
Localized necrosis (the necrotic area < 1/3 of the cross-sectional area)	4
Extensive necrosis (the necrotic area < 1/3 of the cross-sectional area)	5

Table S2 The scoring criteria of auricular appearance

**Grade of appearance**	**Score**
Range of discoloration	
0%	1
1 – 25%	2
25 – 50%	3
51 – 75%	4
> 75%	5
Level of darkest color	
No change	1
Purple	2
Light brown	3
Dark brown	4
Black brown	5

Table S3 The scoring criteria of auricular angiography

Grade of devascularization rate	Score
0%	1
1 – 25%	2
25 – 50%	3
51 – 75%	4
> 75%	5

Table S4 The scoring criteria of auricular arterial histopathology

Grade of histopathology	Score
Occlusion of central auricular artery	
None	1
Partial	3
Complete	5
Damage of perivascular tissue	
No damage	1
Infiltration of inflammatory cells	2
Tissue thickening with proliferation of granulation	3
Localized necrosis (necrotic area < 1/3 of the cross-sectional area)	4
Extensive necrosis (necrotic area > 1/3 of the cross-sectional area)	5
Damage of distal tissue	
No damage	1
Infiltration of inflammatory cells	2
Tissue thickening with proliferation of granulation	3
Localized necrosis (necrotic area < 1/3 of the cross-sectional area)	4
Extensive necrosis (necrotic area > 1/3 of the cross-sectional area)	5

Table S5 Angiography of auricular vein and necrosis of auricular tissue [n(%), n = 6]

Items	NC	EtOH	L-D REI	M-D REI	H-D REI
Auricular angiography					
None occlusion	6 (100.0)	0	4 (66.7)	2 (33.3)	0
Partial occlusion	0	3 (50.0)	1 (16.7)	2 (33.3)	2 (33.3)
Complete occlusion	0	3 (50.0)	1 (16.7)	2 (33.3)	4 (66.7)
Cases of auricular necrosis	0	2 (33.3)	0	0	1 (16.7)

NC negative control, *EtOH* absolute ethanol, *REI* radiopaque ethanol injection, *L-D REI* low-dose REI (0.125 ml/kg), *M-D REI* medium-dose REI (0.250 ml/kg), *H-D REI* high-dose REI (0.375 ml/kg)

Table S6 Pharmacokinetic parameters of ethanol in rabbits of each group

**Pharmacokinetic parameters**	**NC**	**EtOH**	**L-D REI**	**M-D REI**	**H-D REI**
C_{max} (μg/ml, mean ± SD)	1.5 ± 0.9	323.4 ± 197.5	128.8 ± 23.1	257.6 ± 66.2	399.4 ± 57.4
$T_{1/2}$ (h, mean ± SD)	5.8 ± 4.1	1.4 ± 0.6	0.5 ± 0.4	1.2 ± 0.5	1.2 ± 0.8
Vdss (ml, mean ± SD)	0	2259 ± 1476	2415 ± 537.5	1939 ± 1294	1376 ± 1259
Clearance (ml/h, mean ± SD)	0	937.8 ± 484.6	582.7 ± 106.5	1274.0 ± 402.2	898.6 ± 260
T_{last} (h)	8.0	8.0	8.0	8.0	8.0
$AUC_{0\text{-}last}$ (μg·h/ml, mean ± SD)	16.1 ± 23.1	227.0 ± 87.7	74.5 ± 28.8	115.9 ± 22.6	269.7 ± 109.8
$AUC_{0\text{-}\infty}$ (μg·h/ml, mean ± SD)	29.6 ± 36.2	247.9 ± 99.6	111.6 ± 89.8	138.6 ± 58.3	290.0 ± 111.2
$MRT_{0\text{-}last}$ (h, mean ± SD)	4.3 ± 1.5	1.8 ± 0.7	3.7 ± 1.0	1.1 ± 0.6	1.2 ± 0.7
$MRT_{0\text{-}\infty}$ (h, mean ± SD)	11.5 ± 4.5	2.4 ± 1.2	8.1 ± 5.6	2.0 ± 2.4	1.8 ± 2.0

NC group: 0 mg/kg ethanol; EtOH group: 197 mg/kg ethanol; L-D REI group: 79 mg/kg ethanol; H-D REI group: 158 mg/kg ethanol; H-D REI group: 237 mg/kg ethanol. C_{max} maximum concentration, $T_{1/2}$ elimination half-life, *Vdss* volume of distribution at steady state, T_{last} time of last observed concentration, *AUC* area under the curve, *MRT* mean residence time

Table S7 Pharmacokinetic parameters of iopromide in rabbits of each group (mean ± SD)

Pharmacokinetic parameters	NC	EtOH	L-D REI	M-D REI	H-D REI
C_{max} (μg/ml)	1719.0 ± 707.6	1262.0 ± 1083.0	1307.5 ± 747.8	2527.0 ± 1275.0	2382.0 ± 1309.0
$T_{1/2}$ (h)	0.9 ± 0.1	0.9 ± 0.1	0.9 ± 0.1	0.9 ± 0.1	0.9 ± 0.1
Vdss (ml)	138.0 ± 43.6	175.3 ± 81.1	171.7 ± 104.9	138.3 ± 29.3	156.6 ± 65.1
Clearance (ml/h)	136.6 ± 32.9	145.3 ± 34.6	154.3 ± 74.8	144.7 ± 19.2	155.1 ± 42.3
T_{last} (h)	8.4 ± 0.1	8.6 ± 0.5	8.3 ± 0.1	8.2 ± 0.1	8.3 ± 0.1
$AUC_{0\text{-}last}$ [(μg·h)/ml]	1185.0 ± 224.0	1136.0 ± 332.4	2257.0 ± 2935.0	1492.0 ± 195.6	1661.0 ± 460.2
$AUC_{0\text{-}\infty}$ [(μg·h)/ml]	1186.0 ± 224.1	1138.0 ± 332.6	2259.0 ± 2936.0	1494.0 ± 196.3	1663.0 ± 459.6
$MRT_{0\text{-}last}$ (h)	1.9 ± 0.2	1.2 ± 0.4	1.0 ± 0.4	0.9 ± 0.1	1.0 ± 0.2
$MRT_{0\text{-}\infty}$ (h)	1.0 ± 0.2	1.2 ± 0.4	1.0 ± 0.4	1.0 ± 0.1	1.0 ± 0.2

NC group: 156 mg/kg iopromide; EtOH group: 156 mg/kg iopromide; L-D REI group: 184 mg/kg iopromide; H-D REI group: 213 mg/kg iopromide; H-D REI group: 242 mg/kg iopromide. C_{max} maximum concentration, $T_{1/2}$ elimination half-life, *Vdss* volume of distribution at steady state, T_{last} time of last observed concentration, *AUC* area under the curve, *MRT* mean residence time

References

1. ISSVA. ISSVA Classification of Vascular Anomalies ©2018 International Society for the Study of Vascular Anomalies. Available from: https://www.issva.org/UserFiles/file/ISSVA-Classification-2018.pdf.
2. Caraballo C, Desai NR, Mulder H, Alhanti B, Wilson FP, Fiuzat M, et al. Clinical Implications of the New York Heart Association Classification. J Am Heart Assoc. 2019;8(23):e014240.
3. Wang D, Su L, Han Y, Wang Z, Zheng L, Li J, et al. Direct intralesional ethanol sclerotherapy of extensive venous malformations with oropharyngeal involvement after a temporary tracheotomy in the head and neck: initial results. Head Neck. 2017;39(2):288-96.
4. Puig S, Aref H, Chigot V, Bonin B, Brunelle F. Classification of venous malformations in children and implications for sclerotherapy. Pediatr Radiol. 2003;33(2):99-103.
